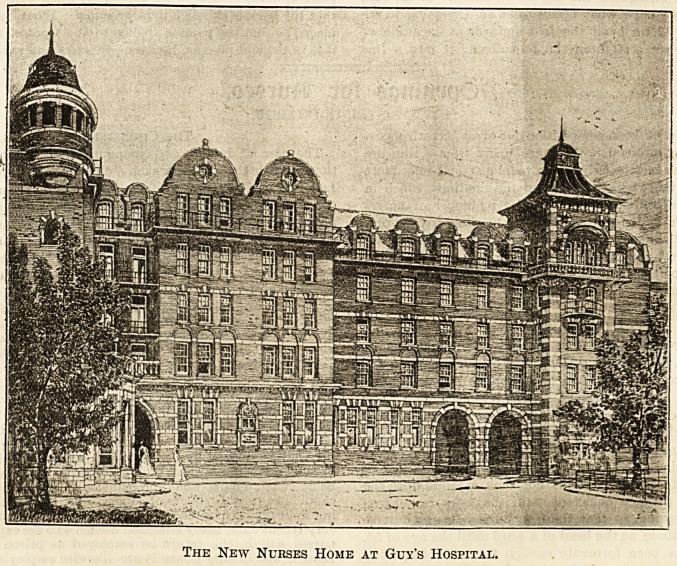# The Hospital. Nursing Section

**Published:** 1902-04-19

**Authors:** 


### The Hospital.

### Nursing Section.

Contributions for this Section of "The Hospital" should be addressed to the Editor, "The Hospital" Nursing Section, 28 & 29 Southampton Street, Strand, London, W.C.

No. 812.—Vol. XXXII. *SATURDAY, APRIL 19, 1902.*

### Notes on News from the Nursing World.

### THE QUEEN AND SOUTHAMPTON NURSES.

It must be gratifying to the nursing staff of the Royal South Hants and Southampton Hospital to know that the Queen has been graciously pleased to intimate the great interest she takes in the efforts to build a suitable home for the nurses in connection with that institution. This expression of Her Majesty's solicitude is made in connection with the munificent offer of Sir Donald Currie to subscribe £2,000 towards the erection of the new nurses' home which is to be built as a memorial to the late Queen Victoria. Sir Donald has also intimated that the Union Castle Mail Steamship Company is prepared to subscribe £500 to the same object upon the understanding that the whole amount required, £5,000, shall have been secured and the work of construction commenced before the coronation of King Edward. It cannot be doubted that this offer, and the knowledge of the sympathy which the Queen takes in the movement, will act as an incentive to the Southampton people to do their duty in the matter without delay.

### ROYAL NATIONAL PENSION FUND FOR NURSES.

The annual general meeting of the members of the Royal National Pension Fund for Nurses will be held at River Plate House, Finsbury Circus, London, E.C. (close to the offices of the Fund), on Thursday, April 24th, 1902, at 4 p.m. Any policy-holder of the Fund who wishes to be present will be welcomed by the Council.

### THE REGULATIONS OF THE MILITARY NURSING SERVICE.

As many inquiries have been addressed io us respecting the regulations of Queen Alexandra's Imperial Military Nursing Service, we may state that they have not yet been formulated. Neither can any definite date be given at present as to when they will be promulgated. So far as the existing members of the service are concerned, the old regulations remain in force until the new are issued. Meanwhile, all applications for admission should be addressed to the Director-General of the Army Medical Service.

### THE WAR NURSES.

The following nursing sisters have arrived from South Africa recently:—On board the *Dunera:* C. H. Potts, A.N.S., acting superintendent, who rejoins the ship; P. M. Carr, A.N.S.R., who requires one month's leave; M. Marions, M. Herring, and M. Meade, all A.N.S.R., and all rejoining the ship. On board the *Roslin Castle:* A. M. Breen, A.N.S.R., who requires one month's leave, and returns to South Africa; and M. Fawcett, A.N.S.R., locally engaged, who requires no leave, and returns to South Africa. On board the *Wakool:* E. L. Warren, A.N.S.R., who requires two months' leave, and returns to South Africa; H. Anderson, A.N.S.R., and S. A. Boneham, A.N.S.R., who require one month's leave, and return (the last returning if required); L. H. M. O'Ryan, A.N.S.R., was invalided home, and requires three months' leave.

### GARDENING OPERATIONS IN SOUTH AFRICA.

The sick and wounded soldiers in South Africa are indebted to some of the nurses for the introduction of English flowers and vegetables in the hospital gardens. In the garden surrounding No. 7 General Hospital, near Pretoria, sunflowers, cornflowers, roses, mignonette, zinnias, and other flowers, grown by Nursing Sister Ferriera, are flourishing, and the same nurse has also, with excellent results, planted vegetable marrows, lettuces, peas, and beans. There is clearly scope for gardening operations in this direction.

### THE SCOTTISH BRANCH OF THE COLONIAL NURSING ASSOCIATION.

At a meeting held last week in Edinburgh a draft of the second annual report of the Council of the Scottish Branch of the Colonial Nursing Association was submitted. It is satisfactory to learn that the sum aimed at in Scotland by Mrs. Chamberlain for her invested fund has been realised, but there is a remarkable contrast between the £797 contributed for that purpose and the £26 5s. which represents all that has been given in the shape of annual subscriptions and donations. The need for an increase of this total must be apparent, and the formation of sub-branches of the Association in Glasgow, Aberdeen, Dundee, and Perth should help to bring in a more substantial sum. An executive committee, with Lady Balfour of Burleigh at the head, has been formed, and a strong desire was expressed at the meeting that there should be a larger number of applications from nurses. It may therefore be assumed that there are openings ready for them. The terms offered to private nurses are £60 per annum, with board and lodging; while for hospital appointments the salaries vary from £70 to £150.

### NURSES IN A PERSIAN HAREM.

The experiences of a member of the Indian Nursing Service, which are given in another column, will be read with interest. In consequence of her knowledge of native languages, she was selected during her probationer's course to accompany the senior staff nurse as interpreter when the latter paid professional visits to native ladies, and on more than one occasion they were received in a Persian harem containing six wives, who seemed extremely pleased to see them. In fact, the impression created by the nurses was so favourable that, to their intense surprise, they were one day invited to resign their hospital appointments and take up positions in the harem—the senior staff nurse as nurse-in-general to the establishment, and the probationer as governess to the only child and companion to all the wives. A very liberal salary was offered, and the polite but decided refusal of it created great disappointment. It did, not, however, break off friendly relations, whereas it is probable, as our correspondent hints, that if the proposal had been accepted, the situation might have become embarrassing.

### NEW HOME AT ASTON.

The nurses at the Aston Union Infirmary may fairly be congratulated on the new Home which has been built for their accommodation. Until its completion the staff, as the vice-chairman of the Board of Guardians admitted at the opening ceremony, were badly housed, and had to put up with many discomforts. In the new building there are bedrooms for forty-two nurses on the top floor, dining-rooms and sitting-rooms of a suitable character, with a room for the superintendent nurse. The cost of the Home, which is not, by the way, entirely devoted to the nursing staff, is about £10,000, and we do not doubt that there will in future be little difficulty in securing the best class of probationers, as the excellence of the training school is not questioned.

### THE RELIGIOUS QUESTION.

A correspondent writing under the *nom de plume* of "Experienced" explains the preference shown at some hospitals for Protestant nurses on the ground that their Roman Catholic sisters require so much special leave in order to attend early Mass or other services. The writer's knowledge is evidently considerable, and we can quite believe that when hospital authorities decline to engage Roman Catholic nurses this is not necessarily done because they dislike their faith. But it is not only Roman Catholic nurses whose religious practices affect the working of an institution. In the case of a well-known hospital in the north of London, the whole of the arrangements were at one period thrown out of gear by the insistence of Protestant members of the staff on prayers at all sorts of odd times. We think that it will generally, if not invariably, be found that in the matter of religious services of any kind, much depends upon the individual, who, whether Romanist or Protestant, if she rightly interprets her obligations as a nurse, will be prepared to cheerfully conform to the regulations of the institution, accepting gratefully such extra liberty as the matron may be able under special conditions to give her.

### A DEFICIT OF £209.

The committee of the North London Nursing Association are face to face with a financial crisis. The twenty-fourth report of the organisation shows that no fewer than 32,873 visits were paid by the nurses last year as against 26,942 in the previous year, an increase of 22 per cent. But, unfortunately, the additional work done has not been accompanied by a corresponding addition to income, and notwithstanding advertisements and special appeals, there is a deficit of £209. In these circumstances the committee have been compelled to intimate that unless more support is forthcoming, it may become necessary to reduce the number of nurses. The fact is not always grasped that the poor of North London, though they do not get the extraneous assistance afforded to those in the East End, and even to those south of the Thames, are very numerous. Still, there must be within the northern limits plenty of persons who can afford to assist in converting the deficit of the Nursing Association into a surplus.

### THE MANGLE AS A RECREATION.

The two delegates from the York Board of Guardians to the Poor Law Conference do not seem to have been delighted with the speeches which they heard in London. One of these, Mr. Snaith, told the York Board, with evident displeasure, that "much was said about nurses and their comforts and convenience, and a good deal more attention was paid to the nurses than to their masters and matrons and their male officers." He thought that "the conference smacked too much of the lady-guardian speakers, and lady secretaries of this-and-that rescue society, and of the parsons;" and he complained that "arguments were carried so far as to advocate ping-pong, lawn-tennis, and a hundred other fads." The other delegate took a similar view, and added that, "as for ping-pong, and that sort of thing, he thought that if the mangle were advocated it would be far more serviceable." The "laughter" which this remark provoked prevents us from feeling much surprise that, as even Mr. Snaith confesses, the accommodation at the workhouse infirmary is inadequate for the nurses at the York Union Infirmary. They, at any rate, have not asked either for ping-pong or lawn-tennis, but they would be very glad if some of the comforts which Mr. Snaith talks about found their way to York. Meanwhile, it is hardly likely that the difficulties avowedly experienced by the guardians in obtaining, or keeping, good nurses will be diminished by the jeering advice that turning the mangle is a pleasant recreation for them.

### SWANSCOMBE NURSING SOCIETY.

The annual report of the Swanscombe and District Nursing Society shows that the number of cases nursed was 93, and that 3,680 visits were paid. It is mentioned that 13 operation cases were attended, and that if there had been no trained nurse available, some of these could not have been undertaken at home. The financial statement is satisfactory, the balance in hand at the end of last year being upwards of £20. Moreover, the investments of the society now exceed £100.

### THE FEDERATION OF ASSOCIATIONS.

A committee was appointed at a conference of representatives of Shropshire families, held at Shrewsbury, to take steps to secure the affiliation of the different nursing organisations in the county. The Bishop of Shrewsbury, in advocating the proposed federation, said that he believed it would be of very great advantage to them all, and would have the effect of introducing the system into places where it did not at present exist. The movement was also supported among others by the Duchess of Sutherland, who suggested the appointment of a superintendent nurse to go about the county to give advice or help, but not to interfere unduly with the local arrangements. Union is strength in regard to nursing arrangements as well as other matters, and we have no doubt that the new departure in Shropshire will eventually prove helpful all round.

### WORK FOR MISS LUMSDEN.

The annual report of the Aberdeen District Nursing Association affords proof that Miss Katharine Lumsden, who has lately resigned the post of matron of the Sick Children's Hospital in the town, but has signified her intention to continue to act as hon. secretary of the association, will have an opportunity of manifesting her characteristic energy on its behalf. The operations of the association are still increasing, but instead of the committee being able last year to wipe off an adverse balance they have slightly increased it, and the excess over income is £81. Miss Lumsden has from the outset freely placed her professional training at the disposal of the organisation, which, in fact, owes its capacity for usefulness largely to her efforts. But now that she has more time at her command she will doubtless address herself to the task of improving the financial position.

### THE TESTIMONIAL QUESTION.

At a recent meeting of the Plymouth Guardians the hospital committee recommended that the superintendent nurse should be given a testimonial, but by 14 votes to 13 the recommendation was rejected. One of the Guardians said he hoped that the board would not commit themselves to a repetition of a state of affairs which, he believed, if brought to the notice of the Local Government Board, would bring reprobation on their heads; while another observed that the decision was obviously dictated by the fact that the nurse was going to apply for another situation. This does not necessarily follow; but, whether it was the case or not, it is a mistake to suppose that the Guardians are under an obligation to give a testimonial to anyone who asks for it, merely with the view of having it ready to produce if required. When a nurse is leaving a post she naturally asks for a testimonial; and if she is a candidate for a particular appointment it is usual, as an act of grace, to supply her with one. But there is no compulsion in the matter, and it is distinctly unreasonable to make the request under any other circumstances.

### THE VALUE OF A JUMBLE SALE.

It was announced at the annual meeting of the Wells Nursing Association that but for the jumble sale, which realised £15, the subscriptions of the members and the collections at the churches would not have been enough to meet expenses. This, of course, shows the value of a jumble sale in such a case, and every possible credit is due to the ladies who worked hard to make it a success. But no nursing association should have to depend upon a chance effort for the wherewithal to pay its way. The Queen's nurse at Wells received 188 patients last year, and paid nearly 4,000 visits. It is therefore clear that her work is appreciated, and there should be no difficulty in securing sufficient regular contributions to cover the cost.

### THE MOTTO OF A NURSING SCHOOL.

The nursing staff of the Parish Infirmary at Portsmouth were recently provided with a small badge to wear on their outdoor uniform, the design consisting of the St. John Ambulance Cross, in white on a black ground, with the initials of the institution in red round it. The same cross is now carried out on their three-quarter cloaks, the cloaks being used as a protection from the cold in the grounds. On Friday, one of the last acts of the out-going chairman was to hang up in the nurses' home an oil-painted and enlarged copy of the cross and motto, "Faithful unto Death," adopted by the school. In his farewell remarks the chairman told the nurses assembled that their calling was most honourable and noble, and that it was, as has been too sadly proved in the present war, sometimes "unto death." He trusted that whatever lay before them they would faithfully carry it out.

### A BAD CASE.

A person named Jessie Bremner, described as "a professional nurse," has been sentenced at Llandudno Police Court to three months' hard labour in the second class for thefts of a most shameful character. The woman, who was convicted of stealing rings from a dying patient, as well as a watch belonging to a lady in another home, thoroughly deserves the penalty she has to pay, and we are glad that the magistrates declined to proceed under the First Offenders' Act. Such robberies by an individual in a position of trust merit condign punishment. But no evidence appears to have been forthcoming at the trial as to the claim of Miss Bremner to call herself a professional nurse. If the people who employed her at Llandudno had taken the trouble to consult "Burdett's Official Nursing Directory" they would not have found her name in it.

### THE ORDERLY AND THE MEDICINE.

In a pretty town of the Transvaal during the present war a field hospital was established, and amongst the occupants of the ward was a man who had suffered several sleepless nights. The doctor on making his evening round told the orderly—a man who in his own small way was not above prescribing for others—to give a dose of nepenthe to the patient, saying, "You will find it on that table," pointing to another part of the room where a bottle of morphia for hypodermic injection and the nepenthe stood side by side. Making a late round, the doctor noticed that the bottles were not placed as before, and said to the orderly, "Has the man had the nepenthe?" "Yes, sir." "What did you give?" "Twenty minims of this," said the man handing the doctor the morphia bottle. "You've done for yourself now," rejoined the doctor, adding, with the intention of frightening the orderly, "There is nothing that I can do now, but if you see the patient's pupils getting very small, call me at any hour of the night." There was no summons in the night. Next morning the doctor came to the patient, and inquired, "How did you sleep?" "Oh, I had a first-class night; slept splendid, sir; but I wish to make a complaint." "What's wrong, my man?" "That orderly, sir, what is on nights, came and stuck his finger into my eye every two minutes when I was asleep, sir." Explanation was, of course, impossible to the patient, but the doctor was satisfied that no more admonition on the subject of promiscuous administration of drugs was necessary in the case of that orderly.

### SHORT ITEMS.

The War Office authorities have presented Mrs. J. Crow Richardson, Glanbrydan Park, Carmarthenshire, with a silver war medal and badge in recognition of her voluntary services on the South African nursing staff during 1900.—The district nursing branch of the Leicester Institution of Trained Nurses benefits to the extent of £1,000 under the will of the late Mr. T. H. Downing.—Miss Lucy C. M. Noble, of the Army Nursing Service Reserve, returns to South Africa in the s.s. *Nubia* on duty.—The following members of the Army Nursing Service Reserve were discharged from hospital to duty in South Africa for the week ending April 5:—Sisters Rosa Lawless, Jessie Moore Craig, and A. E. French.

### Lectures to Nurses on Anatomy.

By W. Johnson Smith, F.R.C.S., Principal Medical Officer, Seamen's Hospital, Greenwich.

### LECTURE XV.—THE JOINTS.

We have seen that in the skull the different bones, both of the cranium and of the face, come into direct contact, and are closely joined together, the lines of union being indicated by the sutures. This mode of junction of bones constitutes an *immovable* joint, or, as it is called by anatomists, a *synarthrosis.*

Between this form of joint and that in which the bones are freely movable as, for instance, in the shoulder and elbow, we meet with an intermediate form in which the opposed bones are united by "buffers" of cartilage allowing only a limited range of movement. Of this mixed form of joint, or *amphiarthrosis,* a good example has already been presented to us by the invertebral discs in the spinal column.

The most developed and typical form of joint is that in which the articulating ends of the bones move freely within a bag or capsule of pliant fibrous tissue. This form, the best examples of which exist in the limbs, presents several

Fig 35.—Section of hip-joint.

variations, all of which are classed together under the common heading of *movable or diarthrodial* joint.

Let us now consider one by one the several elements of a joint of this kind in a diagrammatic sketch of the hip (fig. 35).

1. *The Osseous or Bony Element* (B).—The extremities of the bones taking part in the joint are composed of open or cancellous tissue covered by a thin layer of compact bone. The cancellous tissue, particularly in young subjects, is soft, loaded with marrow, and abundantly supplied with blood. This portion of a long bone is usually in close proximity to an epiphysial line, and is a region which, as it is one of the growing parts of the bone, is endowed with much vital activity.

2. *The Cartilaginous Element* (C C).—The articular or "joint" extremities of the bone are each covered by a layer of cartilage, a bluish-white material commonly known as gristle, with very smooth surface, and tough and slightly elastic. This layer of cartilage when sound and healthy is closely united with the layer of compact bone on which it rests.

*Ligamentous Element* (L).—The cartilage-covered surfaces of the articulating bones are held together by a continuous capsule of fibrous tissue closely adhering to the margins of the cavity or depression on the one hand, and on the other to the neck of the bone below. This fibrous capsule, which is very evident in the joints of the shoulder and hip, varies in thickness at different parts and on different aspects of the joint, and is traversed by well-defined bands or cords of thickened fibrous tissue, to which the name of *ligaments* is specially applied. The fibrous tissue forming the capsule and the special ligaments is made up of straight interlacing fibres, and, though pliant and flexible, is extremely tough and resistant.

*Synovial Element* (S).—The inner surface of the fibrous capsule is lined by a thin and transparent membrane known as *synovial membrane,* which is lubricated by a very viscid, oily fluid like the white of egg, sometimes called "joint-oil," but which we must learn to call *synovia* or *synovial fluid.* The synovial membrane forms a continuous lining to the fibrous capsule, but, except in the fœtus, is not spread over the cartilages.

Knowledge of these anatomical facts will help us to appreciate several points of clinical interest, presented by injury and disease of joints.

The cancellous and spongy tissue at the articular extremity of a bone, being in a young subject highly vascular, and taking an active part in the growth and development of the bone, may be regarded as a "tender" region, and when irritated by undue and too active movement of the limb, or bruised in consequence of some injury, may become a favourable soil for the settlement and breeding of the minute organisms of disease. Thus we might explain the frequent origin of articular tuberculosis or so-called white swelling of a joint, in the cancellous tissue of one of the bones.

The articular cartilage ulcerates sooner or later in severe and prolonged disease of the joint, and the exposure of the inflamed and sensitive cancellous tissue consequent on partial removal of its cartilaginous covering is held to be the chief cause of the painful "startings" of the affected limb, so often complained of by the subjects of joint disease. In advanced age the cartilages become thin, dry, and occasionally very hard, and as they are no longer smooth and yielding the joint is stiffened. Articular cartilage is involved also in gouty affections of a joint, and in advanced cases may be extensively impregnated with urate of soda or gouty deposit. In a violent injury to a large joint, a piece of cartilage may be broken off and thus constitute one of the varieties of loose or floating body in a joint.

The thin and hardly distinguishable synovial membrane is the seat of the many forms of articular inflammation known as *synovitis,* which may be acute or chronic, and the result of injury or disease.

The discharge of "sticky" synovial fluid from a recent wound over a large joint is a grave symptom, as it shows that the wound is a deep one extending into the articular cavity.

The typical joint represented in our diagram whilst preserving its constituent elements of bone, cartilage, ligament, and synovial membrane, undergoes several modifications in different articular regions. In some of the movable joints, those of the carpal and tarsal bones for instance, there is but a slight range of gliding movement, whilst in the shoulder and hip joints, particularly in the former, the corresponding limb can be moved freely in all directions. In other joints, the knee and ankle for example, there is a simple angular movement forwards or backwards. The hand is not only capable of being moved forwards and backwards, but can also be inclined sideways. We have thus *gliding or simple arthrodial joints, ball and socket or enarthrodial joints,* and *hinge or ginglymoid joints.* There is one form of movable joint in which one bone alters its relation to its fellow bone by undergoing a rotatory movement, working either as a ring around a pivot, as in the case of the atlas and axis in the cervical portion of the spine, or, like the neck of the radius, as a pivot turning within the

LECTURES TO NURSES ON ANATOMY.—*Continued.*

ring. If we place the hand palm upwards on a table, we are able by this rotatory movement of the radius to turn the hand over and to expose the back of the extremity without moving either shoulder or elbow. If we carry out a like movement on the skeleton we shall see that, whilst the head of the radius simply rotates around its vertical axis, the lower part of the bone is turned forwards over the ulna. These two joints, the atlanto-odontoid and the superior radio-ulnar, are called *rotatory joints*.

In the upper limb, when the arm, forearm, or hand, or all these segments together are moved forwards the movement is called *flexion*, and when moved backwards *extension*. Thus in bending the forearm we flex it, in straightening the forearm we extend it, and the same with the hand. In the lower extremity, when we bend the knee we flex the leg, when we straighten the leg we extend it. In the case of the foot we have what seems to be an exception to the above order; if we bend the foot upwards at the ankle we extend it, if we straighten the foot—bring it into a line with the leg—we, in an anatomical sense, flex and not extend it.

If a limb be moved outwards away from the middle line of the body—that is to say, if the arm be raised from the side of the chest or the lower limbs be separated—it is *abducted*, if moved inwards towards the middle line it is *adducted*. In the hand abduction and adduction imply deviation from, and approximation to, a vertical line drawn through the middle finger. Thus the thumb is abducted when separated from the fore-finger, and adducted when its tip is applied to the tip of the middle finger. In the foot the fixed line is drawn along the second toe.

There are two other movements which occur only in the forearm as a result of the rotatory movement of the radius on the ulna; the hand when resting on a table with the palm upwards is said to be *supinated*, when turned over so that the back is exposed it is *pronated*. When the hand is placed so that the palm is applied to the front of the chest it is in the mid-position between *pronation* and *supination*.

### Openings for Nurses.

BY A SISTER-IN-CHARGE.

In this age of keen competition for every lucrative post in the nursing profession, there are many women who are likely to suffer increasingly as the years go on; I refer more especially to those who have passed the age-limit, which in many cases is fixed at 35, and to a large number of nurses who, through various circumstances, became paying probationers at the outset of their career, and now find themselves in a most undesirable position, owing to the lack of a three years' certificate. This qualification is now generally insisted upon as a *sine quâ non* in the credentials of any applicant for a responsible post upon a hospital staff.

### The Question of Age.

With reference to the first disability, I would like to suggest that, knowing something of the difficulty experienced by Poor Law guardians in obtaining qualified nurses for their sick wards, it seems as though considerable scope remains for older nurses in this branch of the profession. True, the work is not exciting, and cannot awaken such keen interest as hospital work, because there must ever be a large proportion of chronic cases in a workhouse infirmary; but, on the other hand, there is not such a strain upon the nerves, and, given a sufficient staff, the work is not particularly fatiguing, so that there is no reason why a nurse may not continue as the head of a ward until the age of 50, unless she has been fortunate enough to secure the post of superintendent nurse, which might be held even longer. The wisdom which results from long experience could be used for the benefit of probationers, and the Guardians would be saved considerable expense in the matter of advertising. This last consideration might even result in the offer of higher salaries to capable educated women, and in that case benefit would accrue both to nurses and Guardians. To women who love nursing for its own sake, the care of the bedridden and helpless should appeal strongly, for though it seems at first sight very humdrum work to tend a ward of impotent folk, it is really a triumph of nursing skill to prevent bedsores and ensure cleanliness under what are often very difficult conditions.

### Maternity Work.

Another point which is often overlooked by nurses is the wide experience of maternity work which may be gained in workhouse infirmaries. Any nurse with an average amount of brains may secure the L.O.S. certificate while continuing her ordinary work in the wards, as opportunities are usually afforded for seeing the required number of cases, and of course the theoretical work may be done in off-duty hours. It need hardly be said that the L.O.S. increases the marketable value of every nurse who holds it.

### The Children.

The care of infants and young children, with all the ills they are heir to, especially such a class as are found in Poor Law infirmaries, offers valuable experience to nurses and should qualify them for such posts as matrons of orphanages, industrial schools, and for the superintendence of the "scattered homes" which are now becoming a special branch of Poor Law work. The success which has so far attended the separation of workhouse children from ordinary workhouse surroundings, with a view to giving them a chance in life free from the pauper taint, has been so great, that in the near future, every workhouse of any importance will require thoughtful, conscientious women to take charge of its children, and nursing experience will then be of considerable value.

### Other Openings.

The second disability alluded to is a very serious drawback to obtaining the higher posts in hospital or infirmary work. But there are other openings for nurses in various institutions where the experience and discipline of hospital life may be turned to good account. Inebriate Homes for Women are being opened in all directions and other things being equal, a nurse would probably be preferred above other candidates for this sphere of labour. Prisons are also opening their doors to trained nurses, and as this movement is of very recent date, many educated women who are experienced nurses will in the future be employed as prison matrons. District and County Councils are likewise employing nurses as health missioners in the scattered villages remote from the great centres of population. A knowledge of hygiene, and ability to lecture on home nursing, and especially the management and feeding of infants and young children, are the necessary qualifications for this department of work. In public and private schools nurses are finding employment as matrons; the office practically combines attention to the minor ailments of children, the care of linen, charge of stores, and frequently supervision of servants.

### The Value of Housekeeping Knowledge.

A nurse who has a practical knowledge of housekeeping should be able to command a good salary as a housekeeper in some public institution, or as nurse-housekeeper in a private family; in short, there are plenty of openings for capable experienced nurses, even though they may have passed the age limit and may lack a three years' certificate, and it is for the benefit of such nurses that these suggestions are offered. Let them turn their attention to some sphere which is not so overcrowded as the purely nursing profession, and they will find it not at all difficult to lead happy, useful lives outside the walls of a hospital, whereas if they continue to try and attain the impossible, they will waste both time and money.

### The Nurses of Guy's Hospital.

### THE NEW HOME AND THE PRELIMINARY NURSING SCHOOL.

By Our Commissioner.

It has already been announced that the Prince and Princess of Wales have consented to open the new Nurses' Home at Guy's Hospital, and though when I saw the matron the other day she expressed no definite opinion, she considers it extremely likely that the event will coincide with the annual prize distribution—an arrangement which would have many advantages and render both functions memorable in the history of the institution. The Home is undoubtedly sufficiently advanced to render it easy to complete the work by the beginning of July. Thanks to the courtesy of Miss Swift, I have had the opportunity of inspecting it throughout, by one of the fire escapes, which are on either side. No doubt when it is entirely finished and furnished throughout, the home will look its best, but it was quite possible for me, with the illuminating comments of the matron, to realise its merits. As to particular features, by reason of its size, the dining hall is entitled to precedence. It will seat from 160 to 170, 15 or 16 being accommodated at each table. From a little gallery above visitors may see the nurses at meals without disturbing them, and, in fact, without being perceived. Next in size is the general reception room, which, with its cosy corners, niches and two fireplaces, is exceedingly attrac-

The New Nurses Home at Guy's Hospital.

under her auspices, and am thus able to give the readers of The Hospital full particulars of a building which, alike on account of its size, its construction, and its appointments, cannot fail to be of interest to the nursing world in general, and to nurses who were trained at "Guy's" in particular.

### The Features of the Home.

The exterior, of which an excellent presentation is given on this page, is handsome and imposing, but no money has been spent in unnecessary decoration. When the building material has been swept away there will be an open space with seats. Even in the interior the primary aim has clearly been utility, though a very proper and successful attempt has been made to combine the useful with the ornamental. The entrance hall and principal staircase are worthy of the building, and the dining hall and reception room are of noble proportions. We started our tour of inspection in the basement, where there is every conceivable accommodation, and terminated it on the top floor, ascending part of the way tive. When the settees, sofas, piano, or pianos, writing tables, and screens are in position, and the floor, which, like all the sitting rooms, is of oak blocks, is adorned by Oriental rugs, the effect will be very pleasing. There is on this floor a sitting room for the use of visitors to nurses, which will contain small tables and settees. On the ground floor also is a large classroom, which will include a museum. There are two other classrooms. The total number of sitting rooms for sisters and nurses is no less than fifteen, each of them being of fair size.

### The Swimming-Bath and Bedrooms.

Before alluding to the bedrooms, the swimming-bath and gymnasium claim attention. This is on a very considerable scale, and proves a splendid adjunct to the home. As the matron informed me, it will be used by the nurses three days of the week, and by the students of the medical school the other three. While the home is connected with the hospital buildings by a subway, there is a separate com-

### THE NURSES OF GUY'S HOSPITAL.—*Continued.*

munication from the home and the medical school buildings to the swimming-bath. Nothing, perhaps, would give a better idea of the size of the building than the statement that there are 215 bedrooms. These are all for nurses. Servants will not sleep in the nursing home, but in the existing dormitories in the hospital. The bedrooms, which are warmed by radiators in the charming corridors, are uniform in size. There is hot and cold water in every room, and, when furnished, each will contain a wardrobe, a chest of drawers, a toilet table with marble top, a bed-side chair and table. On the floor there will be linoleum with bed-side mats, and a picture-rail will be provided. To every nine bedrooms there is a bath-room attached. There is a box store and lift to every floor; a separate boot and shoe lift to the boot-cleaning room and a linen shoot into the laundry. The entire top floor of one wing is intended for sick quarters, and there is ample accommodation for seven nurses, including a capacious convalescent room, with balconies all round, from which fine views may be obtained.

When we had concluded our survey I asked the matron who would be in charge of the nursing home.

"A home sister and a dormitory sister," Miss Swift rejoined.

"You mentioned that no servants will sleep in the home. What about the culinary arrangements?"

"I am going to try the experiment of employing men in the kitchen."

### The Financial Position.

"The origin of the home was a munificent contribution by Mr. H. L. Raphael?"

"Yes, it is called after his wife the 'Henriette Raphael Nurses' Home.' Four years ago he gave £20,000 for the purpose, and since other members of the family have contributed generously. Mr. H. H. Raphael and Mr. Walter J. Raphael have given the swimming-bath, at a cost of £4,070. Their condition of the gift was that the money should be devoted to that purpose. Mr. Herbert Raphael has given £1,050 towards the furnishing. The entire cost of the home will be about £60,000, excluding the site, which was purchased by the hospital authorities some years ago, and of this half has been subscribed."

### The Need of the Home.

"In any event you would have been compelled to erect a home?"

"Certainly we should. At present all the nurses are sleeping in dormitories at the top of the medical and surgical buildings, and, what is worse, in an unused ward, which in the public interest ought to have been opened long ago. Only the sisters have separate bedrooms. The remainder are cubicles, divided and curtained off. There are two small sitting-rooms and a dining-hall, which is very badly wanted as a servants' hall."

"What portion of the nursing staff will remain in the hospital?"

"The whole of the 240 attached to the hospital will reside in the home, except the ordinary sisters, who have a sitting and bedroom adjoining the wards, and 20 probationers who live in the matron's house. The private nursing staff, 90 in number, already have a home of their own, with which is connected a district nursing home and midwifery training school."

"When was the new home commenced?"

"Three years ago. The architect is Mr. J. H. T. Woodd. When the Prince and Princess of Wales visited the Hospital recently, they inspected the ground and first floors, and expressed themselves very much pleased with all that they saw."

### The Preliminary School.

"The Home will also afford you the opportunity of carrying into effect the scheme for a preliminary nursing school, about which I should be glad to have full particulars."

"I have just had the proof of the regulations. A preliminary school for pupils has, I have long felt, been a great need here; and in future all applicants for the post of probationer for one year's or three years' training in the wards will be required to pass a satisfactory course of instruction and practical work in the preliminary nursing school, the course extending over a period of six weeks."

"What will it comprise?"

"Tuition and practical work in elementary anatomy, physiology, hygiene, dispensing, bandaging, the making of dressings, the use of instruments, bed-making, with housework, sick-room cookery, etc."

### Number of Pupils and Rules.

"How many pupils do you propose to take?"

"There is accommodation in the home for 15. You have seen their quarters on the third floor—a bedroom each, a common sitting-room, and a cookery kitchen. Their dining-room is over the dining hall, and the class-rooms and museum are for their benefit. Two sisters will devote the whole of their time to their instruction and practical training."

"And the fee for the course?"

"Six guineas, which includes board, residence, tuition, and practical work. The preliminary probationer has to provide herself with indoor uniform, and to pay for her personal laundry. Unless I exercise the option, which I reserve, of terminating the engagement at any time, in which case the due proportion of fee for training will be refunded, no part of it will in any circumstances be returned. At the end of the course it will rest with me to determine whether the probationer shall be allowed to continue her training. There will be an examination; but, of course, no kind of certificate."

"Will the preliminary probationers be allowed to go into the hospital."

"Not without special leave from me. If they want to go into the rooms of the sisters or nurses they will also have to ask my permission. The six weeks' teaching is absolutely additional to the period of training, and the preliminary probationers for six weeks will simply be lodgers in the home."

"Have you made any regulations?"

"I have only so far drawn up a time table. They will be called at 7, on duty 7.30, breakfast 8.30, prayers 9.30, dinner 12.15, tea 5, supper 9, prayers 9.30, in rooms 10, lights out 10.45. They will be off duty one and three hours on alternate week days, and for the whole day on alternate Sundays."

"One more question, Miss Swift. Have you had many applications for admission into the preliminary nursing school?"

"So many that the difficulty will be to choose the most suitable. Other things being equal, preference will be given to those of the best physique. I am quite sure, however, that it is most important that, instead of girls going straight from their homes into the wards, they should go through a course of instruction in a preliminary school, and I intend ours to be self-supporting."

### To Nurses.

**We invite contributions from any of our readers, and shall be glad to pay for "Notes on News from the Nursing World," or for articles describing nursing experiences, or dealing with any nursing question from an original point of view. The minimum payment for contributions is 5s., but we welcome interesting contributions of a column, or a page, in length. It may be added that notices of appointments, entertainments, presentations, and deaths are not paid for, but that we are always glad to receive them. All rejected manuscripts are returned in due course, and all payments for manuscripts used are made as early as possible after the beginning of each quarter.**

### A Visit to the Leper Hospital, Pretoria.

BY AN ARMY RESERVE SISTER.

Through the courtesy of Mr. von Gernet, the medical officer in charge—the same doctor who will always be gratefully remembered by the English for his kindness to our men for many months prisoners in Pretoria—we were at last able to accomplish a long-anticipated visit to the Leper Hospital. It lies about seven miles out of the town on a stretch of veldt immediately below and under the protection of West Fort, looking like a small village of cottages and terrace-houses, with its church and hospital blocks, and higher upon the slope the quarters for the matron and overseer, each standing in their own grounds. Indeed, the Leper Settlement, Pretoria, which is the headquarters for the treatment of this horrible disease in the Transvaal, is larger than many of the towns which have figured prominently in the war—Rustenburg, Machadodorp, Belfast, etc.—and contains some 100 inhabitants. It stands on a large extent of land, a good deal of which is under cultivation for produce for hospital consumption, and some other is turned to good account by the patients for vegetables, which they are allowed to send to their relatives of happier circumstances. The hospital grounds are partially bounded by a low range of hills on one side, and a steep kopje on which stands the above-mentioned fort—one of Pretoria's historical defences. Behind, on the left, winds the long road leading away to civilisation and companionship and the great outside world—a veritable road to Paradise as it must seem to the outcasts of the Leper Asylum.

### The Division of the Settlement.

The Matron, Miss Whiteman, who had most hospitably invited us to come any day, took us over the settlement. It consists, roughly speaking, of three divisions—that assigned to the hospital proper, the houses of the Dutch, and the Kaffir location. We were glad to hear that there were no English patients. A small garden is allotted to each house, in many instances splendidly cultivated and bright with all kinds of flowers, the richness of the soil producing beautiful blooms; the stoeps of the terrace houses were gay with pot-plants, ferns, and cactii, and, climbing up on trellis work, grenadillas, with their delicious fruit, combined the beautiful with the useful.

### Clean and Cheerful.

We had a good view of the interior of many of the houses—homes of leper families. They seemed to consist each of two rooms, a front and back, the stoep, as is so usual in South Africa, being used as a sitting-room. All was delightfully clean and tidy. We came across one particularly sad case—a woman who had evidently enjoyed a good social position, and had travelled in nearly all parts of the world. She, her sisters and daughter, father and mother, were all stricken with the same malady, and debarred from all congenial society and intellectual privileges, which to cultured minds are almost a necessity. In the same row we saw a bright young girl of 16, ironing, and a touch of homeliness was added by a sewing machine. The most pathetic sight of all was a little child of seven, with the face of a woman of 40, but capable of receiving, with childish glee, the 6d. which the matron put in her hand. We were much impressed by the cheerfulness of the lepers. One Kaffir woman, whom we at first thought looked sad and sorry, laughingly contradicted the assertion when matron asked her if it were so. One boy we saw had become totally blind, and had lost both feet; he looked wretchedly ill, but was sitting up on his bed in a comfortable little room.

### Kaffir Lepers.

The Kaffir location was quite clean, an unusual phenomenon with natives, and though not so well furnished, and the rooms frequently flagged with stones instead of wooden floors, the beds were adorned with gaily-coloured blankets, the perfection of beauty in a Kaffir's eyes. Here, too, we found music, and a concertina and mandolin which we were told were skilfully played by two of them—in fact they formed the band at some of the numerous dances held in the large recreation room which we afterwards visited. One fine young fellow, evidently keen for a holiday, had donned his old clothes and reported himself sick; his looks belied his words and did not convince Miss Whiteman, and a dose of rhubarb, much to his disgust, was the unexpected result. Vessels used by the Kaffirs have to be placed outside the houses as a guarantee of cleanliness, each man keeping his own room in order.

### The Patients and the Matron.

Among the Dutch patients was an old man who was insane as well as leprous. He had developed a passion for sweets, and Miss Whiteman told us that a promise of some was sufficient to call down blessings on her at all times, places, and under all circumstances; but any failure on her part to remember provoked as forcible maledictions. It was pleasant to note the respect and pleasure evinced by all the patients at the matron's round, the Dutch acknowledging her by bows, and the Kaffirs saluting and saying "good-day" in the native tongue. It meant more, too, than ordinary courtesy, as the whole settlement is under the sole charge of the matron and overseer (the doctor living in Pretoria) whose control of the 100 patients, including Kaffirs, out on the lonely veldt is entirely a moral one. Though a first-rate shot and able to defend herself, the contemplation of such an emergency for the matron impressed one with the power of character over numerical strength. A native policeman guards the Kaffir location to prevent desertion and is stationed at the gate of entrance to the Dutch part of the settlement.

### Education and Recreation.

Schools for both native and Dutch children are provided. We saw on a slate the young attempts of a little Kaffir at writing. Epidemics of other diseases than leprosy are not unknown and the contingency has been met by very complete arrangements for isolation and disinfection. Dances are frequently held in the large recreation room already referred to, and much patronised by the patients, whose prejudices or convictions are not offended by such a pastime. The custom prevailing at these gaieties differs somewhat from the usual social amenities of a dance. Directly the music strikes up, the gentlemen seize the nearest ladies, *nolens volens*, and rush off in the giddy whirl; if by chance one damsel should happen to be appropriated by two partners "might is right" and is literally the arbitrator, each man pulling his best and the stronger securing the prize! The matron is always present during some part of the evening, showing not only her sympathy with, but her approval of, the patients' pleasures.

### A Strange Church.

One of the most striking buildings in the settlement was the church—the strangest one I have ever seen. It is divided into two parts—a small pathway, literally in the open, making two separate blocks — the smaller comprising a flight of stairs and a pulpit, in which the "predikant" officiates, and the other and far larger half built much in the style of a students' amphitheatre in an operating room, being reserved for the lepers; the straight side of the crescent, if a line were drawn from point to point, being entirely open to the air and bounded by the pathway. The roof also affords much ventilation, not resting on the walls, but raised a foot or two above and supported by pillars.

### A VISIT TO THE LEPER HOSPITAL, PRETORIA—*Continued.*

These precautions are taken to minimise infection for the "predikant."

### Protection for Visitors.

Visitors are allowed to see their leper friends on Sundays. A special building has been erected for the purpose. It consists of four small rooms, two on either side of a passage, for patients and visitors respectively, and across this they converse. The penalty of being "suspect" of leprosy is so heavy—seven years of isolation—that no precautions can be considered too rigorous. We saw two little maids, children of a leper, who were being detained in the settlement, though away from all communication with the patients, for seven years. They were employed in the matron's house and looked the picture of happiness, donning their best bibs and tuckers for our particular benefit; a Sunday bonnet also found its way into week-day publicity on the same occasion—feminine instincts beat true in all parts of the earth.

### The Labour Question.

Government cooks for the settlement, though, if desired, family cooking is allowed. Labour in the grounds is also encouraged in those patients who are fit, and is paid for by the authorities. In two years' time, the matron said, she hoped all the ground immediately around the houses would be under cultivation and look as pretty as it already promised to be in some parts. The present asylum has only been completed three years and took the place of a much older hospital standing a little distance away, which, owing to its insanitary condition, was burnt to the ground by the Dutch.

### The Matron's Quarters.

The matron is furnished with a charming little house of six rooms, built on a high slope overlooking the whole settlement, with views in the distance of long expanses of veldt. Her life is a busy one, as the three rounds made as part of the daily routine, and often supplemented by particular visits, involves an amount of walking that can only be understood by experience. The issue of clothing and all stores is under her control, and secretarial work forms a part of her duty.

### The Course of the Disease.

We did not see much of the disease, but were told that all lepers had sore feet, the extremities being usually the first part attacked. One old Kaffir woman's face had the appearance of much tatooing in raised weals; some had their faces and hands covered with a medicinal powder. Enlargement of the nose, or its total abeyance, seemed too very common. Twelve years is theoretically assigned for the course of the disease, though many patients live on to an indefinite period and die of exhaustion. We heard that the wives of lepers are allowed, if they wish, to come and live at the settlement. One very interesting fact we learnt, that in the event of a leper marriage, any child of the marriage if removed from its parents immediately after birth is supposed to be quite free from infection. Deaths in the settlement are not numerous. A cemetery and two mortuaries round off completely its inhabitants from all other fellow creatures and give an impressive finality to their isolation. Cremation seemed to us a more suitable ending in all respects, but has not yet been adopted.

### Five Thousand Lepers.

Afternoon tea on Miss Whiteman's stoep brought a most interesting visit to a close. Although able to converse fluently in Dutch and Kaffir, English conversation and change of thought was a boon she told us; and one could realise that it was so, and also feel unutterably thankful for immunity from a disease which, while leaving them an existence, shuts off its sufferers so completely from life. It is computed that there are some 5,000 of these in the Transvaal.

### Sponging in Typhoid Fever.

### EXAMINATION QUESTIONS FOR NURSES.

The question was as follows: "If in a case of typhoid fever the doctor ordered sponging of the entire body, how would you proceed to carry out his orders?"

### First Prize.

First take and record the temperature and give some nourishment. Remove the top bed-clothes and hang the sheet before the fire, leaving one blanket over the patient. Place a blanket over the under sheet, or over the mackintosh and draw sheet and upon this a mackintosh the full length of patient, covered by another blanket; this may be done by carefully rolling the patient from side to side. The blankets used for sponging should never be replaced on the bed, but should be specially set apart for the purpose. Having removed the pillow and night-dress (in a recumbent position), hang the latter (or one to replace it) by the fire. Sponging should then commence, and may be with either warm, tepid, cold or reduced to ice-cold water, with or without disinfectant, vinegar or eau de Cologne, but should the evacuations be involuntary disinfectants should be used. The temperature of the water is usually ordered by the doctor in attendance, and to insure accuracy on this point test with a bath thermometer. If the temperature is left to the nurse, tepid water is generally used. Keep the patient covered as much as possible and commence sponging from the face downwards, taking each arm separately, the chest, then the abdomen, the legs being done in the same manner as the arms, while for the back the patient should be gently turned on one side. Plenty of warm, dry, soft towels should be used, and each limb dried separately after sponging. Cold or ice-cold water is generally ordered in cases of high temperature or hyperpyrexia, and this must be frequently changed as it soon becomes warm from the heat of the patient's body; if possible have two sponges, using them alternately.

A nurse should watch for any change in the patient's condition during cold sponging, as a sudden drop of temperature or collapse may occur, in which case the sponging must cease, and stimulant be given to increase the patient's warmth as soon as possible. Hot bottles should always be at hand. Unless ordered more frequently, it is usual to sponge typhoid patients twice a day, or when the temperature is over a given height. To reduce very high temperatures, each limb or part should be repeatedly sponged with plenty of water, particularly the axillæ, palms of hands, feet, and back. Whilst the patient is still lying on the side, the under blanket and mackintosh may be rolled up and out; a warm, dry blanket should then be placed over the one covering the patient, and the damp one withdrawn, thus leaving the patient between dry blankets. The back may be rubbed with spirit, the night-dress replaced, blankets removed (taking care to keep the patient covered), and the bed made in the usual way. The temperature should again be taken and recorded with the sponging, and milk or diet ordered given, and the patient left quiet to induce sleep. The nurse should then disinfect her own hands.—Anglican.

### Second Prize.

Protect the bed with a mackintosh-sheet, over which place a blanket, put another blanket over the patient. Remove the bed-clothes and shirt. Put a hot-water bottle to the feet during the process of sponging. Sponge with tepid, cold, or iced water, whichever may be ordered. The addition of a little sanitas, vinegar, or eau de Cologne to the water is beneficial. Sponge from above downwards, passing the sponge slowly and gently over each part before drying; the rest of the body being carefully covered with the blanket. Dry with a soft warm towel. Commence with the face and neck, then proceed to the hands and arms only uncovering one limb at a time. Next the chest

### SPONGING IN TYPHOID FEVER—*Continued.*

and abdomen, best done by raising the blanket with the left hand and using the sponge with the right; by so doing this part of the body need not be uncovered. The thighs may be sponged in the same way. The legs and feet, as the hands and arms. (Special attention should be paid to the axillæ, palms of hands and feet, as sponging seems to have more effect in these places.) Turn the patient on his side and sponge the back and shoulders under the blanket in the same manner as the front of chest and abdomen. The reason for leaving the back till the last is that whilst the patient is on his side, the mackintosh and under-blanket can be rolled up, and so be easily removed as he is being placed on his back again, and in this way avoid unnecessary movement. Put on a warm shirt, withdraw covering blanket and replace bed-clothes without uncovering patient.

"Anin."

Answers this Month Reach a Good Standard.

Taken as a whole the papers this time are very good, more practical, more condensed, and less discursive. "Anglican" wins the first prize because she grasps the whole subject better than any other competitor. "Anin" is nearly as good, and takes the second prize. "Anglican" mentions what few others do, the necessity of frequently changing the water used, so as to keep it at the same temperature. If one basin of water is used for 10 minutes (however cold) on a patient in a high state of fever, it would certainly no longer be cold. She also says that a thermometer should be placed in the basin if tepid water is used, so that an uniform temperature is maintained. "Anin's" paper is superior in one item, that dealing with hot bottles. She wisely says, begin with one at the feet. Prevention is better than cure. Patients in such a critical state easily lose their vitality, and collapse comes often with terrible rapidity; guard against it, rather than endeavour to fight it when it comes. It is not quite clear why "Anglican" wants to put a superfluity of blankets under her patient—one over the mackintosh is quite enough.

Honourable Mention.

This is gained by "Poppy," "Nanfans," and "Nurse Hancox." The last-mentioned speaks very sensibly of the advisability of always sponging towards the heart, but her paper is too slight to gain a prize.

Question for April.

Give examples of the different kinds of enemata, and state the circumstances under which each kind is most frequently ordered by doctors.

Nurses in our Colonies and Abroad and Elsewhere.

Owing to the repeated efforts of nurses in distant lands to participate in the advantages offered by our examination system, it has been decided to arrange for them a competition twice in the year. The questions will be inserted in April and October, and, to economise space, the rules will be identical with those for the monthly candidates, except that, instead of the notice referring to the competition being open for 15 days, competitors must especially note that their papers are required to reach the editor by September 20th and March 20th. Papers arriving after these dates will be disqualified for consideration.

Question for October.

State what arrangements you would make for supporting a patient with the least possible fatigue during treatment which consists of immersion in a bath for many hours at a time.

The Examiner.

### Visiting Native Patients in India.

BY A SISTER.

As I had acquired a thorough knowledge of both Mahratti and Hindustani, I was picked out during my probationer's course to accompany the senior staff nurse as interpreter in her professional visits to native ladies, and have several times visited them as a staff nurse by myself.

A Persian Harem.

Amongst other homes which we went to was a Persian harem, which then contained no less than six wives. On the days appointed for our visits a closed carriage was sent for us; but the first day there were "tatties" (thick mats) at the windows, which it took some arguing with the coachman to induce him to remove. The house was occupied entirely by the women-kind, the men living in another building altogether. They consisted of the owner of the harem, his younger brother, and a nephew. A short flight of broad, stone steps, led to a very thick wooden door, carved magnificently; this gave entrance to a small hall with large folding doors, apparently only opened to admit chance visitors such as ourselves. The hall opened into a very large drawing-room, divided at one end by a long carved screen, seemingly to form a small dining-room. On either side of this long room were bedrooms, each with a dressing and bath room attached. The whole was furnished to a certain point in English style; the walls of the drawing-room were hung with huge mirrors, there were cosy arm-chairs and sofas, with soft velvet and satin cushions, also a piano. The bedrooms, etc., were fitted up in the same way, with every necessary adjunct; but the absence of an Englishwoman was shown by the fact that there were no draperies nor ornaments, the gilding of the beautiful mirrors was tarnished and broken, and the piano-cover was in rags. In many of these native homes, if they belong to the wealthy, there is a lady-companion or a governess, and it falls to her share to see that the house is properly furnished and kept in order. All the wives wore the Oriental dress, but a few of the younger ones had cut themselves a fringe, and the result was very odd. As we were the first Englishwomen they had ever seen, we presumed that the fringe was the outcome of some fashion magazine, which they would no doubt think it grand to imitate, even in this small way.

A Gathering of the Wives.

We were admitted into the house by a very repulsive-looking eunuch, numbers of whom strolled through the rooms at will; there were also several ayahs. On our arrival, all the ladies came into the drawing-room, and it was quite a difficulty to prevail on the patient to go into the private room to be attended to, so eager were they all to hear news of the outside world. The eldest wife could speak Hindustani fluently; the others understood it, but could only speak indifferently. One would imagine that there would be frequent disagreements among them, but as far as I could judge, they seemed to live in perfect amiability together. The "Bébé," as they called the eldest wife, was held in great respect, and her will was law apparently. There was only one child, a boy between eight and nine years old, very nice looking, with thick, silky curly hair and lovely eyes; naturally he was dreadfully spoilt.

An Offer of Employment.

I often visited this harem, and one day the ladies were strangely uneasy, and notwithstanding all my efforts, I could not remove the constraint. At last it was explained; the "Bébé" said that she had a plan to propose, which was no less than the following astounding proposition :—That the

### VISITING NATIVE PATIENTS IN INDIA.—*Continued.*

staff-nurse and myself should resign our hospital appointments, the former to become nurse in general to the establishment, and myself governess to the child, and companion to them all. The salary offered was liberal, and they were for a while very hurt when we both, without a moment's hesitation, refused the honour. However, after having carefully explained to them that, though we were earning our living from sheer necessity, yet we loved the profession we had embraced, they seemed to understand, and I was pleased to find that it made no difference in our friendly relations. When the staff-nurse and her patient returned to the drawing-room, lemon-tea, sherbet, and cigarettes were handed round. All the ladies of the harem smoked; it was quite a surprise to them to find that comparatively few English ladies indulged in the habit. They begged us to wear "mufti" whenever we visited them; the staff nurse always wore uniform as she visited simply in her medical capacity, but I varied my dress as much as possible.

### Diagnosing a Case under Difficulties.

Before we began our visits the civil surgeon had been called in, but as he comically put it, it was not exactly easy to diagnose a case, when all the examination permitted was the pulse, and a tongue thrust through a hole in a purdah. In spite of his being by no means a young man, he was allowed to make no further examination. The post of nurse in general, had one of us accepted it, would have been a very onerous one, for the wives were full of fancies, and in the case of any real sickness, to give satisfaction the cure would have needed to be immediate; while if the treatment had not been entirely such as they approved of, they would have absolutely refused to conform to it. But as it was I thoroughly enjoyed our short period of intercourse.

### Beyond the Seas.

### DISTRICT NURSING IN SOUTH AUSTRALIA.

Rather more than seven years ago, when I was in South Australia, I had the opportunity of accompanying one of the first two district nurses in Adelaide on her rounds, and of seeing the work which had just been begun in one of the poorest outlying parts of the city. The very words "district nurse" had in those days a foreign sound to many people but that there was need for the nurses was plainly shown by the fact that cases of enteric fever, and various diseases requiring constant attention, were by no means rare. The nurse's life very soon became a busy one.

### The Nursing Society.

Last year, being again in Adelaide, I attended a crowded and enthusiastic annual meeting of the District Trained Nursing Society, the work of which has been during the last seven years of inestimable value to the poorer classes of South Australia. The society is now fairly established, and is likely to go on extending the work indefinitely. There is a general committee of 20 ladies and 20 gentlemen, and at present there are ten districts, each with a local committee and a nurse who has had at least two years' training. Private hospital-trained nurses are accepted for the work, and also nurses trained at the children's hospital. To understand the reason for this one must see or know something of the really good training to be obtained at these institutions. Nowhere have I seen the refinements of nursing, the "little touch" which makes all the difference to sick people, more assiduously practised than in the Adelaide Children's Hospital, and one or two private nursing homes, the matrons of which are excellent women who have had a thorough training in London, or at least English general hospitals.

### All Australians.

The superintendent of district nurses informed me that the nurses are all Australians, but that there had been three English failures! One could only come to the conclusion that the failing must have begun the other side of the world. It is certainly useless for any but good, fairly strong, well-trained and refined nurses to attempt district work in South Australia. The people are thoroughly awake and discriminating, and they look for all-round fitness rather than for mere length of training. It may interest some nurses to hear that the superintendent, who is also secretary, does only holiday work in the districts, and that the holidays are now three weeks for the first year, and a month for the second year and afterwards. The work is kept up by private subscriptions and donations, collections being made in each district by the local committee. The nurses receive £70 a year after the first three months (for three months they are paid at the rate of £65 a year). After the second year the salary is £75. Nursing homes are not fairly established yet, and the nurses find board and lodging, or receive not less than £30 when they live in a home supplied by the society. It is hoped that the salaries will increase; but at present all concerned seem to be satisfied with the wonderful progress which has been made, and no doubt various questions, including that of remuneration, will come under discussion later on. Each nurse is provided with a bicycle which is used only for emergencies. The climate is against constant cycling and it has proved to be too tiring in the hot weather. Two districts are now in happy possession of pony traps, which the nurses fully appreciate, and as savers of time and strength they are considered to be worth the extra expense.

### The Maternity Home.

The seventh annual report of this society gives ample testimony of the good and useful work done by the nurses. They are not allowed to attend maternity cases, as it is thought wise to keep this branch of nursing separate. They are, however, after the lapse of a fortnight, sometimes called in to help the mothers. Not many months ago, during the Royal visit to South Australia, an impressive scene was witnessed by a comparatively few spectators in a large open space in a beautiful suburb of Adelaide. It was the occasion of the laying of the foundation-stone by the Prince of Wales of a Maternity Home, the beginnings of a resting-place for poor women. Under the open sky of that far away land, in the first year of the Commonwealth of Australia, stood the King's son, and near him the son of one of England's greatest poets, the Governor of the State. But it was especially a proud day for Lady Tennyson, for it was her idea, her scheme, this home for which she had thought and planned and hoped, to be devoted to the use of poor mothers in their greatest need. It has also opened up a possibility of obtaining in the future specially-trained district nurses for the women who cannot either leave their homes or obtain admission to the maternity home.

### Presentations.

Hackney Infirmary, Homerton.—Miss Whittington, who has resigned her post as assistant matron of Hackney Infirmary, has been presented with an afternoon-tea service from the nursing staff, a silver-mounted biscuit-box and serviette-ring from the doctors and chaplain. In the presentation great regret was expressed at losing her, and she leaves with all good wishes for her success in her new undertaking as matron at the Small-pox Hospital at Tottenham.

Crumpsall Infirmary.—Miss Haslett, who is leaving Crumpsall Infirmary to take up an appointment as superintendent of nurses at Hull Workhouse Infirmary, has been presented by the matron, on behalf of the nursing staff, with a handsome silver tea-set and hot-water jug, in appreciation of the esteem in which she was held. She was also presented by the maids with a very nice tea-service. She takes with her all the good wishes of the staff for her future welfare.

### Where to Go.

Dowdeswell Gallery. — A sacred picture entitled "Christ and the Little Ones," by Thomas Mostyn, is now being exhibited at these Galleries, 58 New Bond Street.

### The Children's Surgical Ward.

BY AN OCCASIONAL CONTRIBUTOR.

As usual, Eliza Jenkins led the singing, and the surrounding cots joined in, in voices clear or husky, according to the size, age, or disease of the occupant—"Jesus loves me, this I know, 'Cos the Bible tells me so."

Eliza was the eldest patient, her voice was high and shrill; as she sang she beat time on the railings of her crib, so that the curly lamb shook as he stood in his place on the top of the dolls' house.

The voice of Maria Bumpus, on the other hand, was small and gurgling, the words extremely difficult to distinguish, the high notes mysterious and woolly—for Maria was wrapped in cotton wool, of which she was in the habit of taking a mouthful from time to time, and when remonstrated with she would look through the bars of her cot and smile, showing the only two teeth she possessed in the world.

No one had ever seen or known Maria Bumpus in a bad temper. Her temperament was that of the old man on the stile, who lives in the "Book of Nonsense." During the eighteen months of her life she had, like him, "continued to smile." Once only, when her mother dropped her to the floor and broke her hips, had that smile been arrested; but on waking to find herself quite comfortable, with two long wooden legs, and an elephant sharing her cot, she resumed the smile and had worn it ever since.

Her body had become curiously stiff. She could turn her head and wave her arms, her neck was soft and podgy as usual, but immediately under each arm she turned into wood and went on being wood all the way down, as long as she lasted, and for some time after she was finished.

All day and all night she lay on her back. It was dull work at first, until one day she pulled up her jacket and began to investigate her neck and chest. She pulled out great handfuls of wool, tore them to shreds, and dropped them to the floor, refreshing herself with an occasional bite.

After this discovery Maria knew dulness no more, neither did the second probationer, whose work it was to sweep up the debris that lay like snow beneath and around Maria's cot.

Maria had no anxieties. She had been put up when she was admitted, and she remained up ever since. The dressing hour had therefore no terrors for her; on the contrary, she looked forward to it, being very intimate with all the doctors, house surgeons, and dressers.

"Hullo! Maria," they would pause and say, or throw a handful of wool at her, or slip the flat pillow from under her head, placing it on her face, with the elephant on the top to keep it steady, and so pass on, leaving her spluttering beneath.

With Eliza Jenkins matters were very different; she awoke each morning with a feeling of foreboding; gradually, as sounds from the kitchen fell on her ear, she felt a little better, then a delightful smell of bacon filled the air, and, mingling with the waking thought, made it more bearable. When breakfast came, and a mug of cocoa, a piece of fat bacon, and a plate of bread and butter were laid on the toy-board across her cot, she felt distinctly hopeful. Perhaps the tubes in her leg would not require to be moved to-day, and she felt in her locker for her bib.

Eliza was seven, and warranted safe with cocoa or anything else, but sister was more than seven, and a pessimist regarding spots.

With the last piece of bread and butter, Eliza's anxiety returned and night nurse who knew the signs, asked her to hold Maria Bumpus and give her her breakfast while her cot was made.

Eliza cheered up at once, for there was great competition in the ward to hold Maria, and her mornings were generally engaged far ahead.

Nurse, however, took no heed of Maria's engagements, but only of Eliza's face; and when it was particularly long, and she foresaw a bad morning, she would pick up Maria and her wooden legs and her feeder of milk, and lay them down beside Eliza, who would converse with Maria and pour some milk and Mellin's food down her throat from time to time and so forget her woe.

But Eliza knew well that time did not stay, but moved on relentlessly, and took Maria to her cot again, and brought the tin basin of water for Eliza's toilet, followed by nurse with a huge bundle of clean nightgowns and pink flannel jackets; then came temperatures, and thermometers were handed to those patients who could be trusted not to bite the end off. At seven the day nurses walked in, the probationer on Eliza's side passed slowly along washing the lockers; then dusters appeared, and the rack above each bed was dusted. At every step Eliza's heart sank lower. Nurse came with a basket of specimen glasses and dry cloths.

"Here Eliza," she said, "dry these for me or I shall never be done this morning!"

When the ward was swept and garnished, the brass knobs on the cots bright, the doctors' table shining and spotless, Eliza's specimen glasses standing in brilliant readiness, the babies (for the most part a sprightly company) satisfied with their lot, a lot of soap and water, peace and plenty, then the nail scissors went round. They were looked upon as a passing annoyance, and for those who bore the ordeal well there were known to be great rewards. Musical boxes that played "Tommy make room for your uncle," "In the sweet by-and-bye," and "Kathleen Mavourneen" with great rapidity and no pause between, crowing cocks, tea sets, drums, monkeys, and, when Maria could bear to part with it, the elephant; in fact, the general rule was, that Jumbo was the reward of those who had suffered most and borne it best. Thus, when Adam Smith was first admitted, and was sick all day and all night, the elephant never left his arms until the unconsciousness of pneumonia set in, when he crossed the ward to cheer and sustain Thomas William Diggle, who suffered from bow legs and an impending operation on their behalf.

"There's a pretty 'eart," said his mother as they waited in the "out-patients'" on the day of his admission. "E'll set 'ere like a disy while 'is mother goes to buy 'im a kike."

"That I shan't, then," roared the daisy, who had not associated with his mother for two years without profit, and who had trusted to the "kike" dodge before to find himself forsaken, and he struck out with legs which, though bent, were vigorous.

"'Old yer noise, ye dirty crow, or I'll knock ye down," quoth Mrs. Thomas William Diggle, who, finding her wiles thrown away, at once resumed her customary terseness of expression.

After a prolonged wait, Thomas William was borne to the ward, breathing out threatenings and slaughter, and revolving like a catherine wheel in the arms of the probationer who carried him up and "took him in." While his mother, safely rid of him, and relieved of her uncertainty and anxiety on the subject, wiped her eyes on her dirty apron, and "took on" picturesquely, so that the other mothers crowded round. "Go on," they said, "you'll be all right when you've 'ad a drink, an' 'e's a 'ealthy boy, 'e'll be all right."

"Ah!" wailed Mrs. Thomas William, "but they're 'ere teday and gone temorrer.'

### THE CHILDREN'S SURGICAL WARD—*Continued.*

And no one could be sure which part of the remark afforded her most comfort.

When Thomas William had had his bath a beautiful little boy was disclosed to view, with soft, brown, curly hair and big, dark eyes, and when his nightgown was put on, and he was carried to his cot where the elephant awaited his arrival, he would not lie down, but knelt outside, with his hands clasped in helpless misery, and from that moment he was called the infant Samuel.

Eliza Jenkins' worst moment save one occurred at about 10.15, when the nurses came back to the ward with clean aprons on, ready for the morning's work.

The probationer went to the bath rooms and came back laden with porringers, dressing trays, syringes, irrigators, etc.; with these she set the dressings for her side of the ward.

All the while Eliza's gaze was fixed on her with fearful interest. No. 1, happy infant Samuel, wanted nothing, his legs having been straightened and encased in plaster of Paris, beyond the reach of doctors.

Nurse fetched the roll of lint; that, of course, was for No. 2, the burn. Eliza watched the mask being cut, the holes for the eyes, the slit for the nose, the opening for the mouth, the large piece for the chest, the long bandages for the arms, the tiny bandages for the fingers, then the spreading of the ointment on each piece, the folding of it all in a neat pile on a dressing tray, the absorbent wool, the bandages, the syringes, the large blue bottle of solution of soda, etc. No. 2's dressing was carried to her locker.

Then No. 3, Eliza Jenkins; a big dressing tray, some little dressing trays, a large mackintosh, some antiseptic cloths folded neatly inside their pink skins, two syringes, an irrigator hooked to the wall above the crib, a large bottle of warm boracic lotion, a large glass jar of antiseptic gauze, and most terrible and fascinating of all to Eliza, a covered jar of indiarubber tubing.

At about half-past ten, the house surgeons and dressers appeared, and not until then was Eliza's locker complete, for they drew from their pockets fearful brown cases of probes, at the sight of which Eliza wept.

"Oo, oo! don't sir, don't sir," she wailed, but even as she did so, she realized that the moment hanging over her from waking had come, and as she sobbed she unfastened the safety pin and began slowly to unwind the bandages, with hopeless sniffs that could be heard across the passage and down it in the children's medical ward.

The house surgeon was very clever and very kind. "Let's have a look, Eliza," said he; then he took the diet card and food was added, Eliza's being the kind of leg that responded to eggs and bacon.

While the house surgeon worked he talked; he felt with the walrus that "the time had come to talk of many things." His topics were as varied as were those of the walrus. Although he did not actually discourse on "ships, and shoes, and sealing-wax, and cabbages and kings," these seemed to the Nonconformist nurse, who was standing by, about the only subjects on which he did not touch. She handed him each thing as he wanted it, but the severe expression of her face did not change.

Eliza, on the other hand, clinging to the bars of her crib with loud "Oo, Oos," and deep sniffings, could not always prevent a ghostly grin flitting over her wet face, for Eliza liked him; she had given her opinion weeks ago on her admission, and never departed from it. "My doctor 'e's a nice bloke, I likes him."

Each morning towards half-past eleven, when the dressings were finished, Eliza's spirits rose, and the strains of

"Jesus loves me, this I know,
'Cos the Bible tells me so,"

rang through the ward.

The special diets might come up at any moment now. The probationers ran about removing the dressing trays from the lockers, Sister hurried, and directed, from her distant corner by the fire, where Adam Smith's cot stood.

Eliza looked towards her from time to time, and unconsciously lowered her voice. Adam was not doing well, and Sister was bothered. Eliza had intended to play at "Pork and Greens" as soon as the dressing was over, but she saw from Sister's expression that something more devotional and subdued would be likely to meet with success.

The ward seemed to feel Sister's mood. One by one the voices grew fainter and died. Only Maria talked to herself in a young mumbling voice, and the hare-lip baby gave an ominous grunt, which greatly alarmed its special nurse, whose reputation was gone for ever if the grunts continued and the stitches broke.

When a distant rumbling was heard, followed by the clang of tin cans, Maria turned her head to look through the bars of her cot to the door, through which the wardmaid presently passed, carrying the special diets of chicken and fish, followed by the probationer with a tin can of mutton broth. This she placed by a plate of bread crumbs, and at once set about making the babies' dinners. As she mixed, Maria's smile grew broader, and the infant Samuel edged himself and his heavy plaster legs to the side of his cot, the better to enjoy the heavenly vision.

The second probationer came round with the bibs and the feeders of milk, and Sister looked up to see her most stringent rule enforced—a feeder of milk for each child first, and as much mutton broth as could be fitted in conveniently afterwards. But woe to the hapless probationer who did not insist on the milk! All the mutton broth in the world could not wash out this offence.

Throughout the day Sister watched by Adam Smith. The probationer on duty for the afternoon brought her relays of fomentations, smoking hot in the poultice bowl. Adam himself had ceased to take an interest in all the arrangements made for his welfare; he lay on his back, with closed eyes, and wrinkled forehead, his nostrils very thin and transparent, dilating with each breath.

It was very hard work being alive. Sister bent over him constantly, and cautiously poured the minim glass full of barley-water and milk, or raw meat juice, down his throat.

Very few babies slipped through Sister's fingers, so artful was she in fitting the tiny lip of the minim glass in at the corner of the mouth, and getting each of the precious 60 drops down almost by stealth, and without inconvenience to Adam, who was too busy breathing to give her any help.

She watched her opportunity, waiting for the contraction in the throat which meant that the way was clear, for two drops meeting might have dire consequences, there was no strength left for choking.

Round about, tucked into the blankets, were one or two assistants in the fight, hot bottles to keep the cot evenly warm, as Adam was unable to contribute much himself, his body was like that of a thin old man.

Sister had two nests of wool for his heels to lie in, so cunningly made that the very minute heel was supported all round, and rested on nothing; the same plan was followed at all doubtful points, for Adam was made of bad material, and very liable to come through.

His face wore a distressed expression, as of one climbing a steep hill with a heavy burden; once he opened his eyes and looked at Sister, as though he understood they were companions in the fight, interested in worsting a common foe. Adam's mother had been sent for; indeed she was free to come and go as she liked, but for some days she had been engaged in drowning her sorrow, so that her chair for the moment was vacant.

### THE CHILDREN'S SURGICAL WARD—*Continued.*

In the evening a woman carrying a little boy appeared in the doorway.

"Are you the nurse?" she asked of the first person she met. "This is my little boy, 'e's bin a out patient, an' now the doctor 'ave took 'im in; 'is name's 'Enery Hedward." She gave the child to the nurse who did not look hospitable, for this was not the most opportune moment for taking in a new case, just when the ward was being put away for the night. She disappeared with the child, in search of the probationer who was rashly permitting herself to imagine the day's work complete.

The woman left in the ward looked round, and perceiving Sister at the far end, went up to her and whispered.

"What do you say?" said Sister.

"That there young woman went so quick," she repeated, "I was jes goin' to say as you may 'ave some trouble long of 'Enry, with 'im 'avin' neglected of is 'ead somethin' shockin', we never found it out till jes' afore I come, it give 'is pa quite a turn when 'e see it, 'im and me's bin reglir upset."

"How old is he?" asked Sister.

"Well miss, 'e's jes' turned three."

Sister had heard this story before; in reply she called to the probationer.

"Put a carbolic cap on the new child," and turned to Adam without more ado.

As the supper bell rang, a young man sauntered into the ward and seated himself on the table in a friendly way.

"How's the pneumonia kid?" he asked. Seeing Sister's form in the distant shadows he slid to the ground again.

"Evenin' Sister," said he. Approaching Adam Smith's cot, he looked at the temperature chart as he felt for Adam's small bony wrist. Sister was not conversational, so he departed to the other end, where he found the puritanical lady waiting with a wooden face and a resigned air. One minute later and she would have gone off with the others, now she must finish what she had begun, however long it lasted.

"Good evening," said she.

"I smell coffee," replied the house surgeon.

"Indeed," said the nurse. There was a longish pause.

"Where's my kid?" said he, driven to business.

"There," said she, pointing to where the neglectful Henry sat up in his cot, his head swathed like that of Beatrice Cenci.

Nurse crossed over to the cot, and the house surgeon followed. He pulled Eliza's ear as he passed, and paused by Maria to feel for her toe, which occurred about the middle of the cot.

"Please don't wake her," said nurse; but the house surgeon took no notice, having been perfectly aware of Maria's eye fixed on him since he entered.

"Where's the infant Samuel?" he inquired.

"If you mean No. 1, he is in his usual place," replied the staff nurse, who considered the house surgeon blasphemous at times.

After a prolonged examination Henry's diet card was filled up, but the medicine board hanging on the other side seemed so firmly wedged on to its nail that nothing would move it.

The house-surgeon glanced at the nurse, who looked straight in front of her. No help was to be expected from that quarter.

The next moment he attacked the board afresh and wrenched it off, while the nail fell with a loud sound in the sleepy ward. He quickly wrote out some prescriptions and departed, observing "Our work comes to us all in good time if we wait and keep the hammer handy."

The staff nurse wondered for the thousandth time how such a person could expect a blessing in his work.

"His ways are not suited to the sick room," she would remark to the young probationers, who were inclined to think well of him. But she was wrong. Then and afterwards he both expected and received a blessing in his work. He was a great surgeon, wise, witty and tender. He loved and was beloved of every baby he met, and he carried among his other remedies the great healer—fun.

When the children had had their baths and were put away for the night the toys went back to the dolls' house, with the exception of Jumbo, who, in the absence of a more important engagement, slept in Maria's arms.

Then Sister took her last round before reading prayers. From cot to cot she went, considering. She knew the history of each, a ragged lot in body and soul. She knew Mrs. Thomas William Diggle, the mother of the infant Samuel; she was acquainted with the peculiarities of Adam Smith's "Ma"; the unfortunate manners and customs of the household which had produced Henry Edward were too familiar to her, but her hope was inexhaustible, and each night as she came round to her starting-point, the dolls' house, she knelt down and prayed for her household.

There was an occasional movement in the cots as the familiar voice was heard, but no stir among the toys enjoying their hard-earned rest. The curly lamb lay on his side in the shelter of the coal-scuttle, the nigger doll leaned against the Noah's ark, exhausted by the labours of the day, his banjo strapped to his shoulders, his black kid legs appearing through the holes in his old striped trousers, the Golly-Wog hung by his neck, gazing drowsily from the one boot-button eye that remained to him, the trumpets and the fiddle slept on the drum.

Then did Sister pray:—

"Look down, Oh Lord, in mercy upon the children of this house. Make them obedient and humble, pure and truthful. May they remember Thee in the days of their youth, and as they grow in stature may they increase like the child Jesus, in wisdom and favour with God and man."

And so the children slept their little sleep in safety, amid the warm twinkling firelight, heedless of the roar of great London crouching without the walls, in sleepless readiness for its prey.

### Papers for Private Nurses.

BY A SISTER OF A LONDON HOSPITAL.

### Operations.

Probably there is no case to which a private nurse is sent, while she is comparatively new to the work, that causes her more anxiety than an operation case. However well up she may have been in surgical work before leaving her hospital she will be sure to meet a good many difficulties away from the wards. The work varies greatly—often the case will be one of urgent need in some out-of-the-way country place, where it will be necessary to improvise, or dispense altogether with, the very things the nurse has hitherto looked upon as absolutely essential. Or perhaps a case in town where the patient's friends are quite poor, but object to the invalid going into hospital, while on another occasion the house may be in Mayfair or Belgravia, with a titled patient, court physicians and surgeons, and every possible convenience and comfort. The nurse's experience as time goes on will soon show her that each and all have their own peculiar difficulties, and that all call for tact, forethought and adaptability; the power to modify her requirements and to make the best of the circumstances that await her, being quite as necessary as her knowledge of asepsis, and her skill in nursing.

### Emergency Cases.

Often in emergency cases she will not have more than one or two hours in which to make all arrangements, and sometimes far less than an hour. In such a case she will find that neither the patient nor the room has been in the least prepared. The patient, she will be told, has been too ill to be washed or to have the room done; immediately there will come crowding into her mind all the instructions she has had about choosing a room with a good light, having it thoroughly cleaned, everything in readiness away from the patient's view and so forth, and she may be forgiven if for a few moments she feels inclined to run away! In the

### PAPERS FOR PRIVATE NURSES—*Continued.*

necessity for hurried preparations no attempt must be made to sweep or dust the room. If there be time, a wet sheet may be pinned securely over a carpet, or some fresh-cut grass or damp tea-leaves, should either be obtainable, may be sprinkled over the carpet; but it is far better to allow accumulated dust to remain where it is than to disturb it immediately before an operation. Also, if the time be very short and the patient badly needing a thorough washing from head to foot, rather than rushing through the task, give full attention to the seat of operation, and the parts that will afterwards be least get-at-able, and complete the desired end at the first opportunity afterwards, when the process of washing, properly managed, has a very useful place in the soothing and comforting of the patient.

### The Tables Required.

For the room, four tables, or their equivalent, will be wanted. Operation table; table for anæsthetist; table for instruments; table for steriliser and dressings. When there is time, and means allow, it is by far the best to hire a proper operation table—failing this a long narrow kitchen table—but it is not always easily found, and in emergencies the bed often has to do duty for a table. However difficult to manage, should the patient be on a feather bed, this must be removed—no easy task in a little room and with insufficient help. Any small table will do for the anæsthetist. The bed or table to be used for the operation must be placed in the best light obtainable and the small table for anæsthetist put at the head. Another small table will be needed for instruments, and lastly one for dressings and steriliser—the top of the chest of drawers, and the mantleshelf have often served for two tables when I could get nothing else.

### Other Needs.

Bath towels make an excellent substitute for mackintoshes; failing these round towels, of which there are sure to be some in the poorest house. An ordinary meat dish, if possible white and deep, does for instruments, with a cup or small tumbler for Spenser Wells forceps. Two basins for the surgeon, one for his assistant, and one for the nurse are enough. The under part of sponge bowls and soap dishes make very good receivers. Be sure to have a small foot-bath and an ordinary washstand bucket in readiness.

### How to Use the Friends.

The friends will be willing helpers. Ask one to remain outside the room to see that all utensils put outside are immediately emptied, and water-cans or jugs refilled as quickly as possible. Set another the task of having hot blankets ready and coffee made, hot-water bottles filled in readiness to counteract shock of anæsthetic. To give them employment is true kindness; let them feel that you rely on them for help, and you will find how willingly they will do what is within their power. I have often found wives or daughters who were ready to faint and become hysterical brighten up and become different beings when I have pointed out to them that I could not manage single-handed, and have given them something definite to do.

### Towels and Nail Brushes.

The surgeon will bring the instruments and steriliser, probably the dressings and pure carbolic or other concentrated form of antiseptic that he uses, or else, if means and time allow, he may expect the nurse to prepare them, but in such cases he will be sure to give very full instructions. Be sure to have a fire and boiling water; methylated spirit and matches should be at hand. Also have as good a supply as possible of towels. I always carried nail brushes in my bag, for in so many houses they are most difficult things to find in a fit condition to give a surgeon. They are very inexpensive, and can be boiled in readiness for another case.

### After the Operation.

As soon as the operation is over wash the instruments and dry them for the surgeon—he will sterilise them at home probably—then collect all the towels and anything soiled with blood, etc., and put them together ready for removal. Get the room straight as quickly as possible; all this can often be done while keeping watch over the patient if quiet after the anæsthetic. Later on, when the patient can be left to his friends for half an hour, take the soiled towels, etc., to the bath-room and thoroughly rinse them in cold water, getting as much blood out as you can. Then they can be roughly dried before being sent to the laundry. This is the nurse's duty, and how ever many servants there may be, it is one that should never be deputed to them. Of course, when there are two nurses one can sit beside the patient, while the other clears up immediately—a much more satisfactory arrangement, but in emergencies a nurse will often be alone, and there will be no linen "shoot" by which to dispose of the soiled articles. Very frequently in country houses there is no bath-room, in which case nurse must get a washing tub or small bath for the purpose and manage with that. It is wise to carry a catheter and funnel, medicine glass, and small feeding cup—these things are so often needed soon after an operation, and they are often difficult to get in the country. I remember once being told that feeders were quite "out of fashion!" Operations in private houses, formidable as they appear to the patient's friends and to the new private nurse, are not so formidable in reality. Surgeons are generally ready enough to acknowledge the nurse's efforts to have things as she thinks they would like them and to make the best of what has been provided, if they know others are not to be had.

### Avoid Bustle.

A quiet, reassuring manner, tact and thoughtfulness on the part of the nurse, will relieve the patient and his friends immensely; the great thing to avoid is bustle and commotion. On no account talk of other operations; praise the skill of the surgeon if you know anything about him. To carry through an operation calmly and quietly will give a nurse immense power over her patient, and her way afterwards will be easy with his friends; all will look up to and rely upon her, and they will have far more faith in her skill than in one who makes a great fuss and commotion, and who allows them to see that an operation case has power to excite her. Above all, be gentle with the anxieties of the friends, and if possible allow wife or mother to see the patient as soon after as can be, and reassure her as to the present condition and probable result. It means so much to them, and to have a glimpse of the dear one sooner than they expect will often do much to calm their fears, and do no harm whatever to the patient.

(*To be continued.*)

### Wants and Workers.

Would any readers of The Hospital kindly send parcels of white rag to the District Nurse, 59 Oxford Street, Whitstable. It is very much needed for use among poor old chronic patients.

Nurse Drakeford, Ireby, Mealsgate, Carlisle, wishes to thank "A Friend to the Poor" for the kind gift of a bed-rest for the use of her poor patients in this district.

### From Capetown to Salisbury.

### THE ADVENTURES OF FIVE NURSES: BY ONE OF THEM.

We five nurses left Capetown after five enjoyable days at 9 P.M. on Saturday, October 12th. The British South African Agent made every arrangement for our comfort during the long journey, and we took a quantity of provisions to last us six or seven days. We stopped at several stations, and each time had to show our permit. At 11.45 we prepared for the night; none of us slept well, and we were up and having breakfast at 8 A.M. So far, the country wss most uninteresting; nothing but hills of different sizes, and no trees, but the ground was covered with heath of various colours, and small green bushes. In some parts there were masses of purple flowers, rather like Michaelmas daisies, growing in clumps about a foot high. The line is a single one, but there are frequent sidings, where we always found a train waiting to pass, and everyone talks. The soldiers asked us for newspapers, which we did not think of bringing, and the poor fellows were so disappointed; but I gave them roses which had been given to me at Capetown, and that greatly pleased them.

### At De Aar.

On Monday we arrived at De Aar at 2 A.M., and woke up to look at the station, which is an important junction. Here we parted with the Johannesburg train which had kept fifteen minutes ahead of us from Capetown. Starting again at 6 A.M. we reached Orange River station at 10 A.M. where we remained half an hour and bought stamps and post-cards. We crossed over Orange River which is very wide, but there was not much water in it. A little later we stopped at Belmont. The line is well guarded from De Aar; block houses every thousand yards; they are made of corrugated iron with small windows to fire through. Most of them have a dummy dressed up; I saw one standing by a sham gun.

### Modder River and Kimberley.

On Tuesday, about 2 P.M., we got to Modder River, and an officer on our carriage showed us whereabouts the battle was fought. Soldiers are always getting on the train from one station to another, and are glad to talk to us and tell us about things. We reached Kimberley at 4 P.M. Two of us had tea at a friend's house, and we all enjoyed a hot dinner at the station. We were shunted about all the night, and as it was dark, with no lamps, we had to buy candles at sixpence apiece. At 5.50 passengers came down to the train, and all the soldiers got out of the armoured carriage to drink tea on the platform before we started at six o'clock. We had an armoured train in front, with some goods trucks and an armoured carriage at the end of our train. The line was covered with small black beetles near the station, and as they made the rails very slippery, the armoured train could only travel slowly. The Royal Engineers were putting up barbed wire fences, and in many places they have planted great barbed bushes. There are a good many on the veldt, and the new green leaves are just coming out amongst the old white thorns. The blockhouses are still frequent, some with gardens with the name and number of the regiment marked out in white stones.

### A Concert at Mafeking.

We spent Tuesday night at Vryberg. The country is flat and barren, and we saw only Kaffirs. On Wednesday morning there was a little excitement; Boers were seen riding off over the kopje, and soldiers were sent to search a farmhouse near the line. We saw them returning, wheeling a sack in a barrow. They had found a box of dynamite buried under a tree, and a slate in the farmhouse on which the Boers had written the dates of their visits. The officer rubbed out the dates and wrote, "Thank you for the dynamite; we shall find it very useful." We reached Mafeking that night, but it was too dark to see anything of the place. After dinner at the station we ended up with a concert. We left Mafeking early on Friday, and passed through most beautiful country, with high wooded hills, and wild flowers along the line. We curved about through the mountains, as there are no tunnels, and some of the inclines were very steep. We came across swarms of locusts, and had to go very slowly in consequence. Having finished our methylated spirit we could not boil our water for breakfast, but a kind girl at Lopatsi station gave us some Eau de Cologne for the lamp as she had no spirit, and would take no money for it.

### Hunting a Pig.

A little further on, at Crocodile Posts, our armoured train left us, as we were in safe country. The rest of the day was terrible, the heat and dust were stifling. We stopped at a station at 10 P.M. for dinner, and had to drink rain water as nothing else was to be had. About six o'clock in the evening the train was stopped. A pig had escaped out of a crate; several passengers and lots of natives chased it over the veldt, but it would not be caught, and they gave it up as hopeless, and we went on again. We had hardly started, however, when a Kaffir brought the pig up by its hind legs squealing like mad; we drew up again and the animal was put in the van. It shows how casual this country is when they stop the mail train to catch a pig.

### Arrival at Bulawayo.

We reached Bulawayo at 9 o'clock on Friday, October 18th, and were met by a man who conveyed us and our luggage to the Grand Hotel in an ambulance waggon drawn by six white mules and an old Cape cart drawn by two. We had a cold supper and went to bed, thankful to rest in one again. On Saturday we went to see the Civil Commissioner, who made the arrangements for the coach which runs twice a week to Salisbury, and he premised to secure corner seats for us, that we might be as comfortable as circumstances would allow during the three days and two nights of our journey. We had to part with most of our luggage, as very little could go on the coach, and to pack our uniforms into our cabin trunks. In the afternoon we went a drive in a waggonette, like a covered van, drawn by four mules, and we rattled along at a fine pace though the roads were frightfully rough. The country round Bulawayo is barren and dusty, no trees, and very little vegetation. Just as we started we came into a swarm of locusts—pinky-red things, about three inches long; they covered the ground and rose up when we drove into them, looking like a cloud of big snowflakes. I hear that they are called "the African snowstorm." We ended our drive at the Queen's Club, where some sports were going on, and the Police Band playing nicely.

### Stage Coach Travelling.

On Sunday morning, October 20th, we went to church; in the afternoon the waggonette was sent again to take us a drive. We visited the hospital, and the matron showed us round and gave us tea. We afterwards drove to the British South African Police camp, where the officers received us hospitably and showed us the guns, mules, and horses. We returned to the hotel for dinner and went out to hear the band which plays every Sunday evening; we found everyone most agreeable and they wished we were going to stop. On Monday we went to the coach office at 10 o'clock and found a crowd of friends to see us start. The coach held twelve, and eleven seats were taken; it is exactly like the American stage coach, with two seats, each holding three people facing the driver, and two the reverse way. We had the whole of the back seat facing the driver and two other

### FROM CAPETOWN TO SALISBURY—*Continued.*

places back to the driver. Besides ourselves, the other passengers were all men, and some of them were introduced to us: one young fellow had travelled in our train and seemed quite an old friend. We all packed in somehow, and the spare seat was filled with rugs and coats which we should want at night. I cannot help remarking on the extreme kindness of those men, our fellow-travellers; they did everything for us they could think of, and certainly sacrificed their comfort to enable us to get some sleep.

### An Interrupted Song.

At 11 p.m. we stopped to change mules, and we got out for a stroll and ordered coffee. One of our party played her banjo, and we sat outside a little tin house in the middle of the veldt, at 11.30 at night, singing familiar songs to the delight of the men. We were half through "The Old Folks at Home," when the bugle sounded, and we had to bundle back into the coach. On October 22nd, at 6.30 a.m., we stopped at Gwelo; there is a delightful hotel there, and we enjoyed breakfast and a good wash. I felt dazed and giddy for want of sleep, and I fell on to a bed for a little while to recover. Most of the men left the coach at Gwelo, and one woman got in; she had cycled from Bulawayo, and the roads were so rough and dusty, she had been obliged to walk a great deal. The empty coach was fairly comfortable, and we managed to sleep the second night. Part of the country was very pretty, hilly, with plenty of trees; the road was cut through the jungle in some places, and reminded one of an English wood. The inns got worse and worse, and the food funnier; at one place we had tinned salmon and tomatoes and a plate with a dozen boiled eggs and biscuits, for which we paid five shillings.

### Waiting for Mules.

On October 23rd we reached Charter, where we had a good breakfast and nice rooms to wash in. At the wayside shanties the refreshment room is generally galvanised iron, and only circular mud huts for washing rooms. We only went five miles an hour in spite of ten mules, and they were changed every two hours. As soon as the animals were unharnessed, they rolled in the dust. The fresh mules always objected to being brought out of the stable; they all ran away at one place and we had to wait an hour in the night while they were being caught. The mules are kept in splendid condition; they go about as fast as a London omnibus on a smooth road, but a great part of the way they can only walk. We crossed a great many rivers, most of which were dry; we went down the banks of some as steep as the roof of a house; how we got up on the other side I do not know. In the precipitous places we were afraid to look out of the window; it seemed impossible to get down safely. One river near Charter is very dangerous in the wet season; two mules and a driver were once drowned in it, and in another river a crocodile ate a mule. There are two drivers, one to hold the reins, and a head man to wield the whip, which is 30 feet long, and requires some managing. After Gwelo the road is so rough they have a boy, with a short whip, to run along and slash the mules when they go off the road; he hangs on at the window between times.

### Left in Charge at Salisbury.

We got much excited as we drew nearer Salisbury; the country is very pretty and green, and we thought it a great improvement on Bulawayo. It was dark before we arrived, and we could not see much of the town. A pleasant welcome awaited us at the hospital, and we were very thankful to find ourselves at the end of our long and trying journey. The mother superior and sisters who have managed the hospital for some years, showed us round the wards and explained the ways of the place. On the second day after our arrival they took their departure, and we were left in charge of the hospital, and to grapple with the difficulty of managing the native servants without knowing a word of their Kaffir language.

### Not a Typical Patient.

It was while I was working in the eye wards of one of the large London hospitals that I came across "Harriet." She looked a baby of two, and was, in fact, smaller than many children of that age, when she was carried into the ward, with her head hidden on her mother's shoulder, hiding her eyes. Any attempt to open these eyes caused such screams that the doctors in the out-patient department decided that she must come into the hospital and have them examined under an anæsthetic.

She was in reality four years old, and her mother explained that for two years they had kept the room she was in darkened, and she had either crouched in a corner with her chin buried in her chest, or been carried about like a baby, with her eyes carefully shaded from the light. No one could induce her to open them or raise her head; she required more care than the young baby, and at last they had brought her up from the country home to see if anything could be done for her in London; but the mother was much distressed at having to leave her behind. The poor child was put to bed in a shaded corner, and fed and treated like a blind child until the next day when the examination was to take place; the sister, at least, expecting to see the poor eyes a mass of ulcers when the lids were raised.

To the great astonishment of all present in the operating theatre, "Harriet" was discovered to have nothing whatever the matter except the weakness caused by the long continued dwelling in darkness. Doubtless a slight cold and weakness had started it, and the mother had thought she was doing her duty in keeping her little girl in darkness because she shrank from the light.

"Harriet," poor mite, was brought back to the ward where women and a few children were nursed together, and given up to sister and nurses to try and coax her to open and use the eyes that had been rusting for so long.

Her name was the largest part of her, and her most prominent feature was the back of her head, as she kept her face so tightly hidden on her chest. Very gently nurses and patients, who all took an interest in her, set to work to try and coax her back to the ways of an ordinary child. Seated on her cot with her back to the light, at first, and a tempting dinner in front of her, she would furtively peep to see where it was, and then, tightly shutting her eyes, would scramble in her plate with both hands, shovelling most of the contents over the bed. Dolls and toys of all sorts were given her, with the hope of attracting her attention, but all to no purpose; just a peep to be able to clutch them, and then her eyes were tightly shut again.

After a week or two of this the doctor decided that sterner measures must be taken, and poor Harriet's head was to be periodically ducked in cold water, in the hope that the shock would do what gentler treatment failed to. It certainly did. Screaming and sobbing Harriet opened her eyes widely enough, and though, of course, the light was painful to them at first and it meant great patience and coaxing to get her to do more than open them just enough to see that there was no cold water near, they were never tightly screwed up as before.

**NOT A TYPICAL PATIENT**—*Continued.*

In a little while she found pretty things were worth looking at, and Mrs. Winkle, a patient recovering from an operation, mothered her to any extent, constantly trying to interest her and taking her for little walks, by special permission, in the corridor, until at last Harriet became her shadow and trotted after her all day long clinging to her gown, much to the pride of Mrs. Winkle, who, weary with the irksomeness of waiting for an eye to become usable again, was only too glad to spend most of her time over Harriet, a thing of course impossible to the nurses.

The poor child from long disuse had almost lost the use of her legs, and much preferred to squat on the ground, but Mrs. Winkle would not permit it, but trotted her about from bed to bed to pay visits and chatted to her until at last we heard her voice and by degrees she became an ordinary child.

I shall not forget seeing her mother when she came to fetch her, wiping away tears of thankfulness at finding the blind baby whom she had left behind her, fearing some dreadful operation would have to be performed, sitting at the dinner-table, eating her dinner in a precise old-fashioned way and looking round with her pretty blue eyes.

### Novelties for Nurses.

BY OUR SHOPPING CORRESPONDENT.

### MESSRS. GAYLER AND POPE.

(113-117 High Street, Marylebone, London, W.

The region which lies immediately north of Oxford Street is the special home of the medical profession and of private nursing establishments, and the many nurses to whom this neighbourhood is familiar will not need directing to Messrs. Gayler and Pope's establishment in High Street (Nos. 113 to 117), Marylebone, where their requirements and needs have for some time past been specially catered for. But they should make a point of inspecting for themselves the really fascinating materials for uniform dresses, not to speak of cloaks, or minor necessaries in the way of caps and aprons, collars and cuffs, to which this firm is now devoting particular attention for the behoof of nurses in general. There are delightful things in washing dress fabrics, linens, zephyrs, etc., all at very reasonable prices, and in every variety of shade and design. A perfect washing fabric is called the "Wash-well," 6¾d. a yard, 31 inches wide, in most serviceable stripes and checks, and charming colours, and is of sufficient substance to promise good wear. In woollen materials, estamene serges, in useful dark blues and greys, and pretty beiges, in similar colours, range from 1s. 0¾d. to 2s. 11¾d. These can be made to measure, complete, at an inclusive charge of 30s., and 32s. the gown, a price at which no one need grumble, even with the slenderest purse.

Dresses of drill or zephyr can be made to order for 14s. 11d., linen or holland being a trifle more expensive, while the "Wash-well" fabric, alluded to already, can be made up into dresses at the small cost of 12s. 11d. the costume.

Messrs. Gayler and Pope have several excellent designs in out-door cloaks, notably the "Harley," which, made in shower-proof material, with a well-cut detachable cape, very suitable for summer wear, is priced at the reasonable sum of 21s., and in a heavier cloth at 25s. 6d. There are other convenient circular shapes, and a long loose coat, very neat in appearance, will appeal to those who do not care for the more conventional garment. Another attractive cloak is made in a grey cloth, with vandyked collar.

Neat little bonnets, "Princess" shape, with velvet bow and gossamer veil, are another speciality, and very superior, "Sister Dora" caps are to be had at 10¾d. each.

Nurses of to-day are fortunate, indeed, in being able to buy those important items of a uniform wardrobe, aprons, ready-made at comparatively small cost. Linen is much less used than in past years, cotton fabrics have so much improved in make, and though the former is always to be preferred, and really repays a greater initial outlay, for rough work the cheaper aprons now sold fulfil every requirement. The "St. Mary's" and "St. Thomas's" aprons are very good in shape, the former with a square, the latter with a high, round bib, ample, both of them, in the skirt and cut to fully envelop the wearer in the way a properly made apron should. These are made at prices from 2s. 6d. and 2s. 11d. each, costing more if any hem-stitching elaborations are desired, or a specially good and strong linen. We noticed a very pretty cuff, made with a point, and some dainty collars. Foot-gear must also not be forgotten; there are ward shoes in sensible shapes, with noiseless low heels, in glacé and morocco, at 3s. 11½d. and 4s. 11½d. a pair, of which nurses will be glad to know.

Messrs. Gayler and Pope have also a supply of other nursing requisites, clinical thermometers, hypodermic syringes in neat little cases, nurses' wallets, fitted or not, and a variety of other useful adjuncts to a nurse's outfit.

### MESSRS. EGERTON BURNETT.

(Royal Serge Warehouse, Wellington, Somerset.)

Messrs. Egerton Burnett's patterns are more charming than ever, and I can especially recommend for walking dresses their herringbone tweeds, which make up very prettily. The Russian blouse is now such a general favourite that many nurses deciding on a spring dress are sure to select this form of coat and skirt; it is both pretty and useful, and can be made in a variety of patterns with regard to collars and trimmings. For this style, there is an ample number of patterns to choose from, a particularly suitable material being the "Abergeldie," a kind of rough cloth rather like an Irish homespun in appearance, but much cheaper than that delightful material. This is 45 inches wide, and 2s. 11½d. a yard. It is made in various shades of blue, green, grey and red. The "Erin" is the same price and equally nice. For cycling, a material called "the Oval" would be most suitable; it is like men's coating, in greys, blacks and stripes, and looks as if it would never wear out. There are also some white striped flannels which are suitable for tennis or river costumes. There are plenty of other materials in the box, and among them some very pretty fancy ones, from which a good selection could be made, and as I suppose everyone will want to get something fresh for the Coronation, I cannot do better than advise my readers to send to this old-established firm for patterns, among which they will find some particularly pretty washing muslins for blouses, and linens for costumes. As to the materials for uniform cloaks and dresses, everyone knows their good wearing and washing qualities. Egerton Burnett's serges are known all the world over, and I need not dwell on their merits long. To glance through the various galateas is to be reminded of the various hospitals, where "sister" presides over her ward in this coloured dress, and the "pros" fly round in that. But for the benefit of young probationers I may mention that good strong galateas are priced at 7½d. a yard, and that the cloak serges are "waterproofed," and can be depended on for all weathers. It is advisable to choose a second pattern when ordering, as the supplies are being cut from daily.

### CORONATION MEDALS.

For the Coronation most of our gold and silver smiths are producing medals in commemoration of the Coronation. By far the most artistic one I have seen is offered by Messrs. Elkington of 22 Regent Street. On one side of the medal are the crowned heads of the King and Queen, and on the other reverse side is a graceful figure of Britannia leaning on a shield bearing the Royal arms. Above, supported by branches of oak and laurel the Imperial crown is encircled by far-spreading rays, emblematical of the far-reaching sway of the British Empire. In the background is a shadowing of Westminster Abbey, the scene of so many coronations, charmingly introduced. The medals are executed in gold, silver, and bronze, and will be most appropriate gifts to make to our friends over the sea, and to juveniles, as well as an interesting souvenir for ourselves.

### Everybody's Opinion.

[Correspondence on all subjects is invited, but we cannot in any way be responsible for the opinions expressed by our correspondents. No communication can be entertained if the name and address of the correspondent are not given as a guarantee of good faith, but not necessarily for publication. All correspondents should write on one side of the paper only.]

### INSTRUCTION IN HOME NURSING.

"A. F." writes: Some of your readers may be interested to hear of a non-professional nursing movement which has just taken place at a seaside town in South Devon. A number of resident ladies having a praiseworthy desire to become acquainted with the principles of home nursing formed a class among themselves, and, having secured the services of a fully-trained hospital nurse in the neighbourhood, they have been meeting regularly for six weeks in the house of a member of the class, and taking great interest in learning as much as could be taught to amateurs of the rules and methods of home nursing. At the end of this time they all very creditably answered a set of examination papers given them by the nurse who had acted as teacher, and the answers showed them to have acquired practical information which can hardly fail to be of use to them in the cases of more or less serious illness and accident which so frequently occur in most families.

### NURSING IN WORKHOUSE INFIRMARIES.

Another "Workhouse Nurse" writes: In my experience of workhouse life it is not usual for officers to be supplied with extra diet, if considered necessary by the medical officer; there is a list of rations approved by the Guardians; anything extra the officer provides for his or herself, except in special cases, when the Guardians make the allowance; it may be by the medical officer's recommendation, but he cannot order it. For instance, an officer of 23 years' standing was very ill, I may say in a dying condition; the Guardians directed the master (by his own request) to provide anything that was considered necessary or that would do good, which he, being a thoroughly kind-hearted man, did with the greatest pleasure. Another master asked me to go and see an officer who complained of feeling ill, but he suspected was shamming (it was a little way of this master to think people were shamming when they complained of feeling ill). I found the man very exhausted, and beat him up an egg. I asked the matron to give me a teaspoonful of brandy to put in it, which she refused, as the man was not a pauper. The doctor came and ordered brandy at once, but even then the master and matron refused to give it, or even lend it. I sent to the town and bought a bottle of brandy and other things. This officer, in spite of his shamming, died in a fortnight.

### SHUTTING THE DOOR ON ROMAN CATHOLIC NURSES.

"Experience" writes: With regard to the somewhat severe criticisms of "A Lover of Justice" I think it will be found on examination that there are two sides to the question. Theoretically, I quite agree with the principle that, all other things being equal, a Roman Catholic nurse should stand as good a chance of appointment as her Protestant sister, but during my many years' experience in hospitals I have so often seen matrons accused of intolerance for refusing to allow Roman Catholic nurses to leave their work to attend early Mass or other services, that I do not wonder a certain amount of hesitation exists in engaging nurses of that denomination. Another, and what I think is the chief reason for this hesitation is, that as attendance at the various religious services is of very great importance to the Roman Catholic, her Protestant sisters can and do all attend the hospital chapel, she must go to her own church, and unless the services fall in with her hours off duty, which in the case of the early morning is very improbable, special leave has to be obtained. The nursing staff of most hospitals is, as a rule, only just sufficient to carry on the work, and the granting of special leave is always a difficulty, for it entails increased responsibility for those left on duty. Should a large number of the staff, therefore, periodically require this special leave it would seriously affect the working of the institution.

### THE "NURSES' NEVER."

"Fijian" writes: I read with interest "Medical Lecturer's" columns of "Nurses' Nevers" as they appeared in your pages, and also read with surprise the indignant and even rude and un-nurselike remarks which his suggestions caused to emanate from the pens of some of our sisters in the nursing profession. Perhaps it is now rather late to comment on the lecturer's careful and practical hints, but as some of the readers of The Hospital live at the Antipodes, I think distance may be pleaded as an excuse for my delay. I wondered why nurses should feel so irate because a medical man has taken the trouble to put into concise form and easily remembered words a few rules which cannot be anything but useful to us all. Does the fact of being a trained nurse, or one in course of training, guarantee that we are infallible beings? I think not. If we carefully review each day's work in our own minds, many things must occur to us that we might have done, or said, to make a patient more comfortable in body and happier in mind. Are we always as careful in our methods of working as we might be? Away from the vigilant and critical eye of the ward sister, does not a nurse sometimes do her work hurriedly to "save time"? I think "Medical Lecturer" must have personally observed nurses doing some of his "Nevers," and was prompted to send his paper as a help to their memories. While drawing their attention to certain facts which reflect on a nurse's work, he does not fail to enumerate suggestions which are conducive to their health and comfort. In conclusion, I consider it bad taste to criticise and comment rudely on a medical man's work, for no one has a nurse's truest interests more at heart than he, and no one could be a better friend to a nurse provided she is loyal to her profession. One more "Never" I would add to the already complete list, "Let us never be ashamed or indignant when we are told anything by those who know more than we do, and are anxious to teach and help us."

### ACTION AGAINST A NURSING INSTITUTE.

"Jas. Barnard, of the Nursing Institute, Llandudno," writes saying that our notice of this case is inaccurate, and implies so much more to the injury of this Institute, that it cannot be passed over in silence. He continues:—"It states 'Miss Boardman had been wise enough to have a written agreement and she had not lost it.' Every nurse enters into this agreement, which was commended by the judge, while strangely Miss Boardman's plea for its non-production by her was that her copy dated December 9th, 1901, *was lost*! As to the absence of a counter-claim, the engagement was of a temporary nature for the time being, because of negotiations which had taken place between the White Star Company and herself, the result of which Miss Boardman represented to be, she had been selected for the next vacancy; hence, when the agreement was signed, a promise was made to her that she might go within three months from that date without notice. Unfortunately, the agreement was not so endorsed. This omission saved the nurse from falling quite to the ground between the two stools. Judgment without costs, together with the judge's remarks, tell their own tale. The judgment as it appeared in the local and daily papers is enclosed. Let it speak for itself."

[The report of the case upon which our remarks were founded appeared in the *Liverpool Mercury*, and we fail to see either in that report or in the report which Mr. Barnard himself forwards, any allusion to the loss of the document or anything to show that our statements were either inaccurate or injurious to the institute mentioned, the only omission being that we did not state that costs were not given to the plaintiff. Ed. The Hospital.]

### "THE CASE AGAINST HOSPITAL NURSES."

"Adeline Babington Sheppard" writes: Miss Johnston tells us that Ruskin, in criticising a certain picture, said that "there was but one fault which the artist had not committed, he had not drawn the tree with the roots uppermost." Following the analogy of this criticism, may I not say that there is but one failing not attributed to nurses in Miss Johnston's "black list"—that of ignorance and incompetence? It is at any rate assuring to open the account with this much to their credit. It is said that the indictment against the nurses is a "strong" and "formidable" one; but, before inquiring into the charges, let us see who are the complainants. Miss Johnston does not leave us in doubt as to this; "The accuser's name is legion," which she explains to mean "the general censure" of the British Public. In sympathy with the number of prosecutors is the number of charges, and it would probably be difficult to discover in any contemporary literature such a collection of vituperative invectives as are found in the short article under review. Some of these are "offensive," "almost brutal," "inconsiderate," "hardened and indifferent to suffering," "rude," "irritable," "sharp-tongued," "bad form," and "absence of the humane qualities." Surely a certain class in the East End would be grateful for such additions to their ordinary vocabulary! Can anyone question the assertion that the indictment is a "strong" one, or that the charges are correctly characterised as a "black list?" Miss Johnston says "it is easy to bring charges." True! nothing easier. But how about proving such? Is this equally "easy," and if so, how comes it that throughout the article there is not one single particle of evidence brought forward in support of any one charge? Nor is there the faintest indication of any of these having been made on the personal knowledge of the authoress. Miss Johnston adopts another course. *Assuming* her charges to be proved, though she has made no attempt to do this, she is suddenly seized with sympathy for the accused, and changes her rôle from prosecutor to advocate; from the self-constituted champion of a shocked and indignant public, to counsel for the nurses themselves. Even were such advocacy needed, I venture to think that the nurses would hesitate before accepting that offered by Miss Johnston, and I should not be surprised at their calling to mind the old aphorism "Save us from our friends!" Miss Johnston is entitled to her opinion, but I am doubtful whether vague assertions, however well intended, are likely to secure respect. A long and intimate acquaintance with the nursing sisterhood in their various capacities, in hospitals, infirmaries, and private work, satisfies me that, as a rule, they are not forgetful of their responsibilities to their employers, to their training schools, and to themselves. And many are actuated by a higher motive still, remembering Who said, "Inasmuch as ye do it to these, ye do it unto Me."

### GIVING HIS ALL.

"Matron" writes: The following pathetic and true story may, I think, interest your readers:—Phillip is a lad of 13, very small for his age, and with a deformed spine which prevents him from lifting up his head. A rather wide mouth gives almost a grotesque look to his general appearance. He had been with us several weeks when a letter came to him from his home. We asked him if all were well at home and, in reply, he handed us over a long letter from "Mother," telling how she and father had both been ill and not able to work and how money was so short they hardly knew what to do. "Father had an offer of 10s. for your little dog," the letter said, "but we will not sell it unless you say we may, Phillip; father does not even know I am telling you this." Poor Phillip; the little white dog had been given to him by a kind friend, and had got to know him so well. We knew it would be a struggle for him to give it up. "You had better answer mother's letter," I said to him; "I am sure you will do what you feel to be right about it." The letter was at last written and before posting I read it through. Such a bright little letter, and not a word about his being not so well and having to stay in bed. "We have such a nice time here, and there are lots of games to play with. I am very happy. I go out in a bath-chair, and sometimes have tea in the field." At the end of the letter he said: "I shall be very sorry to part with my little dog, but if you want the money for the house, I do not mind your selling it. You may sell the dog" And then in large letters: "I say you may sell the dog." His letter was posted, and with it an order for 10s. and a message saying, "Please keep Phillip's little dog for him, and accept the enclosed towards your house expenses." Phillip heard about it all from his mother a few days after, and his gratitude was most touching.

### SLEEPING IN A PATIENT'S ROOM.

"Dorothy" writes: This question is always a sore point with nurses, and one which it would seem can never be satisfactorily settled. I was interested in the letter written by "Inexperienced," which appeared in The Hospital of April 5th. At the same time I sympathise with her very much. I am a private nurse of a few years' standing, and will mention what I do under similar circumstances. I had a case of hemiplegia only a few weeks ago. My patient was a gentleman [about 50 years of age, and I was the only person who could do anything for him. At first I had to be on duty from 9 o'clock at night till 2 o'clock the next day, then I went out for a walk and went to bed; even then I was not able to get consecutive rest, because I had to be roused once, sometimes twice, to pass the catheter. However, in about a fortnight, I was able to arrange things differently, as the patient got able to do without someone sitting up all night, as, of course, he slept well enough between times. I got the largest room in the house made into a double room (it was a country house, and the room happened to be a very large one). The patient's bed I put at one side of the room and mine at the other. Then I got two screens put along by the front of my bed, so I was quite shut off, and yet if my patient was awake I could hear him. This was the only thing that could be done, as the patient would not sleep alone, and as a nurse his wife was quite helpless. I got up nearly every three hours to pass the catheter, keep the fire up, and so on. I had a small table at the side of my bed with a candle on it ready to light. This I did when the patient called me. Then I got up and put my slippers and dressing-gown on before I came round the screen, and so was as much dressed as I would have been if I had come out of an adjoining room, and also I had almost as much privacy. I had a bedroom for my own use in which I kept my things and used as a sitting-room; I undressed in it before going into my patient's room for the night, and when I got up in the morning I went straight to my own room to dress. The patient's wife sat with him the while. This I did for over four months, and got on splendidly. Of course all my meals I had with the family down stairs. I got the patient out of the bedroom into another on the same floor during the most part of the day so that the sleeping room was well aired and cleaned. I do not think there is nearly as much in "sleeping in a patient's room" as there always seems when the words are used. It depends on the nurse, patient, and last, but by no means least, on "circumstances." It is not wise to do it if one can in any way manage without, but I consider that if the case and surroundings were like this one of mine there is no harm done. The doctor I had said that it was a most admirable arrangement for everyone, and what is a nurse's duty but to please her superior officer? When I have male patients—and I have more male than female—I try to sleep in another room on the same landing if I cannot have one opening out of the patient's room, and in the former case I have one of those portable electric bells, and I find if people have not already got one, they are most willing to get one. They are quite inexpensive. The bell I place in my room, and every night I put the cord along the floor under the doors and up to the patient's bed, where I pin the handle on the pillow to secure it. I find that any patient who is well enough to be left at night can always manage to ring me up when anything is wanted. I would therefore strongly advise "Inexperienced" not to take what may be more experienced nurses' views too much to heart. They may not be placed in the same position. Therefore it is best for a nurse to use one's own discretion in her cases. I always find at most of my cases it is always "When in Rome one must do as Rome does," because all circumstances alter cases, and a nurse has always to bring her mind to suit circumstances. I have been private nursing now in an institute for nearly four years, and find on most points, especially those concerning "sleeping in patient's room," that most of the nurses only agree to differ. If nurses, as a class, would "advise" nurses who are new to private work rather than "lecture" them, it would be a great help and much kinder.

### Appointments.

Central London Sick Asylum, Hendon.—Miss E. E. Scutt has been appointed charge nurse. She was trained at Lewisham Infirmary, where she has since been charge nurse.

Clayton Hospital, Wakefield.—Miss Louisa Strange has been appointed sister. She was trained at the North Devon Infirmary, and the Lincoln County Hospital, where she was afterwards staff nurse.

Crooksbury Sanatorium, Farnham.—Miss Jessie Wells has been appointed sister in charge of the main block. She was trained at St. Bartholomew's Hospital, London, for 18 months, and afterwards for three years at the Birmingham Infirmary. Miss Wells holds the L.O.S. certificate.

Derby Royal Infirmary.—Miss Edith Ullathorne has been appointed sister. She was trained at the Royal Infirmary, Liverpool, for three years, and has since been on the private staff for 15 months.

Dorking Union.—Miss Eleanor L. Chippendall has been appointed head nurse. She was trained at Kensington Infirmary, where she has since been night superintendent.

Fleetwood Cottage Hospital.—Miss Sara E. Barlow has been appointed nurse-matron. She was trained at Stanley Hospital, Liverpool, and has since been sister in charge of children's ward, and theatre sister and sister in charge of the male wards in the same institution. From March 1899 she has been head nurse at Longton Hospital.

Gainsborough Union Infirmary.—Miss Ada Woodthorpe has been appointed head nurse. She was trained at Cork Fever Hospital, and has since been charge nurse at Doncaster Union, Balby, and Sarforth Fever Hospital.

Gravesend Hospital.—The lately-appointed matron, Miss L. Mary Paine, having resigned owing to sudden family bereavement, Miss Bessie Coleridge Davis, one of the other selected candidates, has been elected to fill the vacancy. She was trained at King's College Hospital, where she was also night sister; has since been matron of Bromley Cottage Hospital, home sister and housekeeper at University College Hospital, matron of cancer wing at Middlesex Hospital, and at present is night superintendent at St. George's Hospital.

Hastings Union.—Miss Mary Elston has been appointed assistant nurse. She was trained by the Meath Workhouse Nursing Association at the Royal Hospital, Sheffield.

Maidenhead Infirmary.—Miss Kate Dance has been appointed assistant nurse. She was trained by the Meath Workhouse Nursing Association at St. Lucy's Home, Gloucester.

Stockport Workhouse Infirmary.—Miss E. E. Douglas has been appointed day sister. She was trained for three years at Crumpsall Infirmary, Manchester, and has since for two years held the post of ward sister in that institution.

### Death in Our Ranks.

We regret to announce the death of Miss K. McCowan, who passed away on March 22, at 3.20 P.M., from acute dysentery, at the Refugee Camp, Springfontein, South Africa. Sister McCowan, who was much esteemed by the staff and patients, was trained at the Western Infirmary, Glasgow.

The death is announced, on April 3rd, of Mrs. Martha Bonswell, who was for three years in charge of the scarlet fever ward at the Milton Hospital, Portsmouth.

We also regret to hear of the death of Miss Shirley, Matron of the Staffordshire Institution for Nurses, on Friday last week. Miss Shirley was exceedingly popular with the members of the nursing staff, and will be immensely missed by them at the Home in Stoke. The excellent work which she ably directed has frequently been mentioned in our columns, and it will be no easy task to supply her place.

### For Reading to the Sick.

MY LOVE OF THEE.

Lord God of Hosts most Holy and most High,
What made Thee tell Thy Name of Love to me?
What made Thee live our life? what made Thee die?
"My love of thee."

I pitched so low, Thou so exceeding high,
What was it made Thee stoop to look at me
While flawless sons of God stood wondering by?
"My love of thee."

What is there which can lift me up on high
That we may dwell together, Thou with me,
When sin and death and suffering are gone by?
"My love of thee."

O Lord, what is that best thing in the sky
Which makes heaven heaven as Thou hast promised me.
Yea, makes it Christ to live and gain to die?
"My love of thee."

*C. Rossetti.*

Whatever may be doubtful this remains certain. Every man who loves God a little is loved by Him much more; every man who loves God much is still loved by Him more.

*C. Rossetti.*

The life of love is to be lived just there where the difficulties are thickest; just there where all is hard and perplexing: because it is just there where God would manifest His love. You know that love, how strong, how consoling it is you can show how work can be done, troubles borne, and temptation resisted by a loving and true-hearted child of God. If, indeed, we are to attain to anything like completeness in the spiritual life, it must be by the gathering together of all the elements of vigorous Christian action into that Divine virtue which is to be not an ornament of our character merely, but its whole expression—Love. "The loving soul is meek, gentle, humble, and patient; the soul that is hardened in self-love hardens itself still more" (*St. John of the Cross*, vol. ii., p. 567). And these manifold ways of exhibiting a true love will sustain our own consciousness of God and the delight of His Presence. They will make our sense of the Divine love stronger and more healthful; more reasonable and less emotional. They will make all life glad and beautiful and strong; they will be the evidence of God in us.

The truth of the Divine Immanence is very inspiring, very consoling to the heart that only longs to love worthily. There is a direct appeal from God through all the evidences of His Presence and care in the world, whether we trace them in natural beauties or in the no less wonderful order of human life. He surrounds us with tokens of His Own Presence; He appeals to our hearts unceasingly by the occasions which are given to us when we can speak, and act upon the promptings of His love.

Here is your opportunity for the days to come. Do not wait for the occasion which may never be given; but give yourself wholly, be yourself at once and in everything the one through whom God may manifest His life. You are called to share the Divine Love, to be the one through whom others are to learn its reality and its power. The more you perceive it in yourself, the more inspiring will it become, until life will lose its sadness and be filled with a new delight.—*Rev. J. Brett.*

### A Book and its Story.

THE LAND OF OUR QUEEN.*

Denmark is within thirty-six hours' journey of London, and its inhabitants by race, customs, and religion are nearly allied to us, they share also kindred instincts in their love for the perils and excitement of an ocean life, and in their literature is traced much that is identical with our own, yet to the average Englishman the country is terra incognita. Sweden and Norway are overrun with tourists, Denmark remains comparatively unvisited. The inhabitants bear little trace in the present day of their warrior ancestry, except perhaps in a certain dogged tenacity, which underlies a phlegmatic exterior.

The accomplished author of "Denmark, Past and Present," has lived in the country, studied its history and people, and her book is a particularly interesting and lucid study of a land which should be more known to English people. Its name is naturally endeared to us through its association with that of our beloved Queen Alexandra, "the sea king's daughter from over the sea."

Most readers know that the old Eddas and Sagas of the Vikings are rich in chronicles of our own nation. "The story of the conquest of England by the Danish kings, of the burning of London Bridge by Olaf the Saint, of the discovery of America by the hardy Norse warriors 500 years before the advent of Columbus, and of many a wild foray and fight on our island shores, can never be read with indifference by anyone claiming to belong to the race of the Anglo-Saxon. The greater part of Northern England, East Anglia, which included the Isle of Ely, Cambridgeshire, Norfolk, Suffolk, Essex, Middlesex and part of Hertfordshire, were so populated at one time by Danes and their descendants that they were under Danish, not Anglo-Saxon, law. This was due also to the fact that four Danish kings, from 1003 to 1041, bore rule in England, viz., Svein, Canute, Harold Harefoot, and Hardicanute. (There is a quaint superstition in Cambridgeshire that pulsatilla, or purple pasque flower, which grows about Fleam Dyke, flourishes only where Danish blood has been spilt.) In the vocabulary of Northern England we find many words whose terminations prove their Danish origin; for instance, toft, beck, tarn, dale, fell, haugh or how (hill), and many others. The yeoman stock of England must look to the same source for ancestry, and a very considerable part of the present population of the midland and northern counties may with certainty trace their origin to the Norsemen, and especially the Danes." We must not forget that Shakespeare drew largely on Norse literature for his immortal creations. The ghost in "Hamlet," the apparition in "Macbeth," the fairies in "A Midsummer Night's Dream," and "The Tempest," are painted closely after their Scandinavian originals. The following paragraph is interesting in connection with the above: "Commentators on Shakespeare have been surprised that he should have been so well acquainted with the details of the scenery described in 'Hamlet,' without having visited the country. Light has been thrown on this subject by the discovery amid the archives of Helsingfors, of a curious and instructive document which shows that in 1585 a wooden theatre, in which a troupe of English comedians had been performing, was burnt down. The names of the artists are given, and almost all of them belonged to Shakespeare's company."

Helsingfors is now a flourishing commercial town, with a fine harbour standing at the head of the Sound. Within a rifle shot is the Swedish Coast, over whose gleaming waters the massive towers of the fortress of Cronberg keep sentinel. The hotel at which the author stayed in Helsingfors was a curiously constructed one. The interior resembled a ship ashore. Long wooden passages, with steps up to one room, and down to another, and the bedsteads were those in nautical language known as "standing bed-places." The dining-room had lockers, like a cabin, and mariners were constantly on the look-out through the port-holes with the aid of a telescope. The coast is not considered dangerous, but a pilot stated that he had seen as many as six wrecks in a night. A view of Fredensborg, the summer palace of the Danish Royal Family, shows a plain, unpretentious building, with whitewashed walls, standing round a courtyard. In the centre is a fountain, shaded by trees, and in the background rises a copper-roofed cupola. The picturesque little village with its pond, and shops, and pretty houses, lies at the gates of the Royal residence. Fredensborg takes its name ("Castle of Peace") from the fact that it was here the treaty which put an end to the eleven years' war between Sweden and Denmark in 1720, was concluded. But it has every claim to the title from other reasons. "It lives up to its name. Nothing can be imagined more reposeful than the simple white house, the long alleys of tall trees ending in the enchanting blue of the lake of Esrom, the quaint statues, and the solitude of the park." The Royal apartments are described as being perfectly unostentatious in their furniture and arrangement. "The King's antechamber is a model of frugal furnishing, his audience-chamber measures about 16 by 20 feet. The small dining-room is used when the family are alone, or the weather is too cold to warm the larger one, at other times it serves for the members of the Court. The apartments of Queen Alexandra are three simple little rooms; the first is a bedroom the whole furniture of which consists of two plain mahogany bedsteads side by side, a round table, an ordinary sofa, a few chairs and a dressing-table; of course curtains and ornaments were wanting when I saw the place, but no young girl in our days could be more simply lodged than this royal lady when in her father's house." Denmark stands out among the minor countries of Europe in the number of distinguished men of letters to which it has given birth there. Hans Andersen's name, perhaps, is the most familiar to English ears, but Holberg, Ewald, Oehenschläger, and others are writers of distinction.

In science the names of Tycho Brahe, the great astronomer, Oersted, the discoverer of electro-magnetism, which led to that of the electric telegraph, are those of world-wide reputation. Then the immortal sculptor, Thorwaldsen, was a Dane also.

The primitive character of the country is suggested by the following statements. "The little children go to school carrying their books in a knapsack on their backs; peasants in sabots, servant girls with short sleeves, and old women with curious poke bonnets adorned with lace are characteristic figures. In search of change of food I entered a shop over which 'delikatesser' was inscribed. Upon investigation I found these consisted of dried herrings, cheese, and a little jam." The Danes are singularly polite and good-natured. If you buy stamps, they will stick them on for you; if you want an address looked out, it will be written down; and when you enter a train or omnibus the conductor greets you with "Good day," and bids you "Farewell" when you depart. The chapters on modern Danish literature, folk stories ancient Scandinavian and old Danish literature, are of particular interest.

* "Denmark, Past and Present." By Margaret Thomas. (Publishers: A. Treherne and Co., London. Illustrated. Price 6s.)

### Echoes from the Outside World.

### The Coronation.

Amongst the guests who are expected to be present at the Coronation—at least, they have accepted the invitation of their Lord-Paramount the Emperor of India—are five of the Indian potentates. Foremost amongst these is the Maharajah of Gwalior, whose country is as large as Scotland and Wales put together, possessing some of the most splendid buildings of the world; next comes the Maharajah of Jeypore, who is proud of being able to trace his line directly back to the Sun God, and the capital of whose kingdom, containing 160,000 inhabitants, is famous for being built of rose-coloured stone, so that the houses always appear as if tinged by the rays of the setting sun. Amongst the curiosities is a huge cage of live tigers at one end of the principal street. The Rajah of Kolhapur is the ruler of a province which was once essentially warlike, and is now essentially peaceful, with board schools and many other marks of civilisation. The Nawab of Bahawalpur is proud of the fact that his people—who are nearly all Mahommedans—have always been faithful to the British rule. The Maharajah of Idar takes the place of the Rajah of Nabha. His State is situated in Mahi Kantha, and is under the political control of the Bombay Government.

Tsai-cheu, Prince Ching's son, who has been appointed Chinese envoy to the Coronation, has left Peking on his way to Europe. Another interesting item is that King Lewanika, the Paramount Chief of Barotseland, and one of the most important, loyal, and intellectual natives of South Africa, has been invited by the King to attend the Coronation. He has already left Lialui, his capital, with a few personal attendants.—The chestnut horse "Hussar," to be ridden by the Prince of Wales in the Coronation procession, has been trained in the stables of the 7th Hussars at Aldershot, and has been sent to Marlborough House on completion of its education.

### South Africa.

The burial of Mr. Rhodes on Thursday last week was a simple and touching ceremonial. A hymn having been sung, and a psalm and prayers said, an address was delivered by the Bishop of Mashonaland who read Mr. Kipling's poem amid profound silence. The wreaths sent by the Queen, Dr. Jameson, and the brothers of Mr. Rhodes, and the Rhodesia flag were buried with the body. The Doxology concluded the service. Afterwards the people were permitted to file slowly past the tomb. Many of them carried away small pieces of loose granite as a memento. Three thousand Matabele, under their Indunas, were present at the ceremony. The place of burial is a large stone kopje, so steep and rugged as to be almost inaccessible. The grave, which is cut three feet deep into the solid rock, is encircled by six boulders, and the whole space around it is only 15 yards long. In London while the interment was taking place in the Matoppos Hill, a memorial service was held in St. Paul's Cathedral, the nave of which was occupied by an enormous assemblage, many thousands being necessarily refused admission. Among the congregation were representatives of the King and Queen and the Prince of Wales, several Cabinet Ministers, and other notable persons. At the same hour a memorial service was also held at Bishop's Stortford, the birthplace of Mr. Rhodes.

On Monday Lord Kitchener reported that Commandant Beyers had been attacked in the Northern Transvaal by a British force under Colonel Colenbrander, who accounted for 106 of the enemy. We lost an officer killed and two officers and five men wounded. A patrol sent out from Bultfontein was attacked by a superior force, and lost three killed and 14 wounded. There has also been an engagement in the Western Transvaal, where the enemy attacked Colonel Kekewich's column. The assault was beaten off, and the Boers left on the field 44 dead and 34 wounded. Twenty unhurt prisoners were taken. Our casualties were two officers and five men killed and about 50 wounded. Another serious disaster has befallen a troop train at Machavie. Thirteen New Zealanders were killed and the same number wounded. The negotiations for peace on the part of the Boer envoys are proceeding.

### Foreign.

Serious rioting has occurred in Belgium, and the disturbances at Brussels have been of a most omnious character. The immediate origin of the outbreak is the Socialist campaign in favour of universal suffrage, but other causes have admittedly been at work. The riot at Brussels on Saturday started in the Rue Steenport, where the troops charged with fixed bayonets, and extended to the Rue Haute, the "East End" quarter of the capital. Here the troops had to encounter missiles thrown from the windows, including knives, brickbats, and glass balls charged with corrosives. Three persons were killed, and more than a hundred wounded. The hospitals are filled with the latter. King Leopold presided over a meeting of the Cabinet on Sunday, at which it was determined to resist the demands for universal suffrage. The Socialists advocate a universal strike.

The Minister of the Interior in the Russian Government was fired at on Tuesday afternoon, at St. Petersburg, as he was about to enter the Marinski Palace to attend a meeting of the Committee of Ministers. Two of the bullets struck M. Sipiaguine, and though he was at once carried to the Maximilian Infirmary, and received every possible attention, he died within an hour. He was under 50 years of age, and had risen rapidly from one office to another. His assassin, who offered no resistance to arrest, gave his name as Balschanett, and said that he had been punished for participation in the disturbances last year at Kieff, where he was a student at the University. For his treatment on that occasion he had vowed vengeance against M. Sipiaguine.

### Parliament and Politics.

The Budget was brought in on Monday by the Chancellor of the Exchequer, who announced a gross deficit of £41,000,000. In order to meet this sum, he proposed new taxation to the extent of £5,150,000, a loan of £32,000,000, a draft on Exchequer balances £3,850,000. The new taxation is to be provided as follows: £2,000,000 by an additional penny to the income tax, making 1s. 3d. in the pound, £500,000 by doubling the stamp duty on cheques and dividend warrants, and £2,650,000 by reimposing the registration duty on imported corn and flour which Mr. Lowe took off in 1869. The last impost existed for twenty years, and amounted to 1s. a quarter in the case of corn and 1s. 6d. on flour. It is now to be at the rate of 3d. per cwt. on corn and 5d. per cwt. on flour. Sir Michael Hicks-Beach declared that the impost ought to make no difference in the price of bread, but some of the bakers appear to think otherwise.

### Philanthropic.

On Friday last week Lady Verney laid the foundation-stone of a new Public Library and Village Hall, which is being erected at Steeple Clayden. The cost, about £1,500, is being defrayed by Sir Edmund Verney, Bart. The village adopted the Free Libraries Act, last year, and owing to its success, Sir Edmund is presenting the parish with the new buildings. The following message was received from Miss Florence Nightingale: "So glad the foundation-stone is being laid of the Steeple Clayden Public Library. I do, with all my heart, wish it success, and think a public library is good for body and soul. That God's blessing may rest upon it is the fervent wish of Florence Nightingale."

### Notes and Queries.

**The Editor is always willing to answer in this column, without any fee, all reasonable questions, as soon as possible.**
**But the following rules must be carefully observed :—**

1. **Every communication must be accompanied by the name and address of the writer.**
2. **The question must always bear upon nursing, directly or indirectly.**

**If an answer is required by letter a fee of half-a-crown must be enclosed with the note containing the inquiry, and we cannot undertake to forward letters addressed to correspondents making inquiries. It is therefore requested that our readers will not enclose either a stamp or a stamped envelope.**

*Discolouration over Vertebræ.*

(20) A nurse is anxious to know how you can account for a discolouration of one or more vertebræ of the spine, from apparently no cause. There is no pain or aching, and there is no history of a fall or strain. After a medical examination, there is no trace of spinal caries to be found, though there is a family history of spinal caries. The discolouration still remains after weeks of complete rest in one position. It does not interfere in any way with walking, bicycling, or any kind of exercise.—*E. S. D.*

A slight discolouration over vertebræ which are habitually rubbed or pressed upon is by no means uncommon, and is without pathological significance. It is often met with about the waist.

*Soxhlet Apparatus.*

(21) In the directions supplied with the Soxhlet apparatus for sterilising milk three-quarters of an hour is given as time required for the saucepan to boil freely. The bottles hold 6 ozs. Can you tell me whether that time is too long if the bottles are only half filled with milk ?—*Mary.*

It would be quite against the principles of the Soxhlet apparatus to fill the bottles only half way up. One of the objects of employing separate bottles is to avoid heating the milk in the presence of a considerable quantity of air. The size of the apparatus and the proportions of its various parts may be taken to have been worked out with the object of ensuring that the milk shall be raised to the required temperature, and retained at that temperature for the required time, by following the directions, and any departure from these directions, even to the extent of only half filling the bottles or of using a smaller number of bottles, will be likely to interfere with the results. It must be remembered that the object of the apparatus is not to boil the milk, but to maintain it at a given temperature for a given time.

*Dispenser.*

(22) Will you kindly tell me the best course to pursue in order to qualify as a lady dispenser ? What are the prospects of employment to such ?—*G. C. G.*

Write to the Secretary, the Pharmaceutical Society, 17 Bloomsbury Square, W.C. The prospects are good for women with plenty of brains and fully qualified.

*Queen's Nurses.*

(23) Will you kindly tell me the rules, regulations and conditions of the Queen's Jubilee Nursing Institute ? I should be glad of your advice as to the most remunerative branch of nursing as I have aged and afflicted parents whom I wish to help, and district nursing seems to afford me the opportunity of sharing the home life.—*Jubilee.*

The General Superintendent, Queen Victoria's Jubilee Institute for Nurses, St. Katherine's Precincts, Regent's Park, London, N.W. will give you particulars. (2) District nursing would enable you to carry out your wish of living at home. So would maternity nursing to a certain extent, and a popular maternity nurse with a good connection earns a very fair income.

*Government Appointment.*

(24) I have had two years' training in a school for nurses recognised by the Local Government Board, and two years' private nursing. Please tell me how I can obtain an appointment under the British Government.—*Nurse L. B.*

Nurses eligible for Government posts must have had three years' training in a general hospital.

*Nerves.*

(25) Please tell me where I could write for advice for a young person suffering from nerves and head complaint.—*Anxious.*

You must obtain medical advice. If a fit case for hospital treatment you might take the patient to a hospital devoted to that species of complaint.

*Beef Extractor.*

(26) (1) Kindly tell me how to press juice from raw beef, and say (2) if there is any machine made for doing so.—*Enquirer.*

(1) Mince the beef finely and place in a clean linen cloth, then twist the ends the reverse ways, as for a fomentation ; or, better still, place it, a small quantity at a time, in a lemon squeezer. The juice flows much better if the beef is first cut in cubes and placed for a very short time, just enough to singe the outside, over a hot fire on a gridiron. The lemon squeezer should be warmed. (2) Yes and sold by Messrs. K. and W. Wilson and Son, 90 Wardour Street, W.

*Massage.*

(27) Can you tell me of any place in Manchester where I could learn massage ?—*G. E. M.*

Write to the Secretary, the Society of Trained Masseuses, 12 Buckingham Street, Strand, W.C., and ask her to recommend you a teacher.

Can you tell me where a blind girl could obtain a certificate for massage ? She has been instructed by a trained masseuse and has had several cases, but she is anxious to obtain a certificate if the terms are within her means.—*Sister S.*

See reply to *G. E. M.* The Secretary will advise her.

*Visiting Nurse.*

(28) I should be much obliged if you will give me some information as to the best way of starting work as a visiting nurse. I see that one of your correspondents has sent you a card containing her fees, etc., and I wonder if she would let me see it, as I think that it is a good plan to have the terms printed. I send you particulars of my training ; do you think that it is good enough for the work I propose to do ?—*Convalescent.*

You should call upon the doctors under whom you wish to find employment and give them full particulars both of your training and of the terms which you propose to ask. Any good stationer will help you to draw up a neat card if you give him your instructions. Your training and the terms which you ask seem very fair. If you do not succeed as visiting nurse you might find employment as district nurse.

My sister and I have just been trained in massage, Weir Mitchel, Nauheim, and medical electricity. I have also had two years' general training. We are anxious to get visiting work and to live in rooms, so we would be very glad if you would kindly tell us where we would have the most chance of success ?—*Nurse Ellen.*

You would be most likely to succeed where you are best known. Probably the doctors under whom you were trained would be able to recommend you to patients.

*Book.*

(29) Will you kindly recommend a good book upon the nursing of children. I have a "Chavasse" which is excellent but which omits medical terms.—*Nurse C. C.*

"The Mother's Help and Guide to the Domestic Management of Her Children." By P. Murray Braidwood, M.D. Price from the Scientific Press, 2s.

*Queen Alexandra's Naval Nursing Service.*

(30) Would you kindly give me particulars as to Queen Alexandra's Naval Nursing Service. What are the rules and to whom should candidates apply ?—*F. S. P.*

Apply to the Director-General Medical Department of the Navy, Admiralty, Craven House, Northumberland Avenue, W.C.

*Lecturer.*

(31) Will you kindly tell me what qualifications are necessary in order to become a lecturer on home nursing under the London County Council ?—*A. C.*

You must be a fully-trained nurse and be able to impart your knowledge in a clear and graphic style.

*Concentration Camps.*

(32) Will you kindly tell me where I can obtain information concerning the concentration camps and tell me if it is likely that two nurses holding three years' certificates would be able to get work and good pay ?—*E. R. E.*

Apply to the Under Secretary, Colonial Office, Whitehall, S.W.

*Care of Invalid.*

(33) I should be glad to know what I might reasonably charge for taking care of an invalid going to the Cape.—*S. D.*

It depends upon whether you are merely in charge of the invalid during the passage, or whether you are going to the Cape on purpose, and who pays your fare.

**Standard Nursing Manuals.**

"The Nursing Profession : How and Where to Train." 2s. net ; post free 2s. 4d.

"Art of Massage." (Second Edition.) 6s.

"Elementary Physiology for Nurses." 2s.

"Elementary Anatomy and Surgery for Nurses." 2s. 6d.

"Practical Handbook of Midwifery." 6s.

"Surgical Ward Work and Nursing." Revised Edition. 3s. 6d. net ; post free 3s. 10d.

"Mental Nursing." 1s.

"Art of Feeding the Invalid." 1s. 6d.

All these are published by the Scientific Press, Ltd., and may be obtained through any bookseller or direct from the publisher, 28 and 29 Southampton Street, London, W.C.

## Figures and Tables

**Fig. 35. f1:**
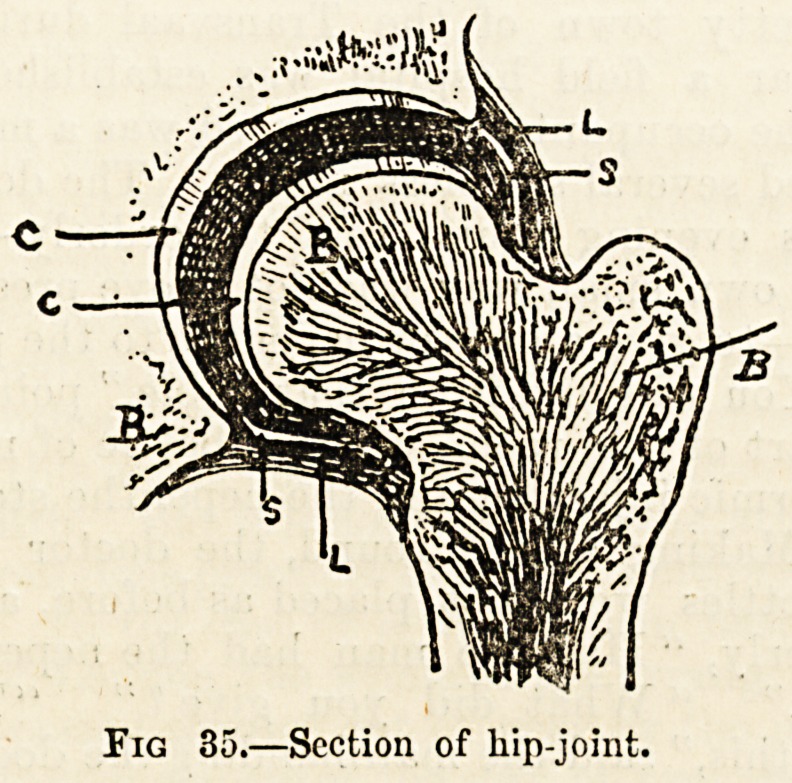


**Figure f2:**